# Exploring the conformational landscape of menthol, menthone, and isomenthone: a microwave study

**DOI:** 10.3389/fchem.2015.00015

**Published:** 2015-03-11

**Authors:** David Schmitz, V. Alvin Shubert, Thomas Betz, Melanie Schnell

**Affiliations:** ^1^Max Planck Institute for the Structure and Dynamics of MatterHamburg, Germany; ^2^The Center for Free-Electron Laser ScienceHamburg, Germany; ^3^The Hamburg Centre for Ultrafast Imaging, Universität HamburgHamburg, Germany

**Keywords:** terpenoids, microwave spectroscopy, conformational analysis, chirality, molecular structure

## Abstract

The rotational spectra of the monoterpenoids menthol, menthone, and isomenthone are reported in the frequency range of 2–8.5 GHz, obtained with broadband Fourier-transform microwave spectroscopy. For menthol only one conformation was identified under the cold conditions of the molecular jet, whereas three conformations were observed for menthone and one for isomenthone. The conformational space of the different molecules was extensively studied using quantum chemical calculations, and the results were compared with molecular parameters obtained by the measurements. Finally, a computer program is presented, which automatically identifies different species in a dense broadband microwave spectrum using calculated ab initio rotational constants as initial input parameters.

## 1. Introduction

Terpenoids represent a large and diverse class of organic molecules, derived from adding substituents to their core building block, the five carbon isoprene unit. Many plants produce terpenoids as secondary metabolites with varying chemical and biological activities (Wagner and Elmadfa, 2003), such as anti-bacterial (Ben Arfa et al., 2006; McKay and Blumberg, 2006; Kotan et al., 2007), antioxidant, anti-inflammatory (Braga et al., 2006) and anti-fungal properties (McKay and Blumberg, 2006; Numpaque et al., 2011). Furthermore, terpenoids account for the largest share of the emission of biogenic volatile organic compounds (BVOC) to the atmosphere (Guenther et al., 1995; Kanakidou et al., 2005; Bateman et al., 2011). BVOCs are subject to oxidation reactions in atmospheric chemistry impacting Earth's climate (Lelieveld et al., 2008). To explain or even predict the molecular origin of a specific chemical or biological behavior, knowledge of the molecular structure, the potential energy surface (PES), the conformational flexibility, and the internal dynamics is required.

Broadband microwave spectroscopy is a powerful tool to study the molecular conformational landscape and molecular structures in detail. Due to their importance in many biological processes, a number of microwave spectroscopy studies of terpenoids have been reported recently. Internal dynamics and the conformational preferences of a linear monoterpene, linalool, was published by Nguyen et al. (2013). Of the monocyclic monoterpenes, perillaldehyde (Avilés Moreno et al., 2009), carvone, limonene (Avilés Moreno et al., 2012), carvacrole and thymol (Schmitz et al., 2014) were investigated with high-resolution microwave spectroscopy. Kisiel et al. studied the structure and dipole moments of camphor using microwave spectroscopy (Kisiel et al., 2003). In the present study, we investigate the gas-phase structures and conformational space of menthol, menthone, and isomenthone based on vibrationally and rotationally cold gas-phase microwave spectra and quantum chemical calculations.

One of the primary advantages of microwave spectroscopy is its ability to obtain information about the gas-phase structure of molecules to a high precision. Nevertheless, for a complete structure determination, rotational constants from numerous different isotopologs are required. An example of such an effort was recently reported for the comparatively large molecule, strawberry aldehyde (C_12_H_14_O_3_) (Shipman et al., 2011). However, this approach remains experimentally demanding and time intensive. Another way to extract structural information from the experimental data is comparison with quantum chemical calculations. The assignment of an experimentally obtained spectrum to a quantum chemically optimized structure (e.g., by comparison of rotational constants, barrier to internal rotation, dipole moments, nuclear quadrupole constants) has given good results, even for larger molecules and complexes (Caminati, 2011).

**Figure 1:**
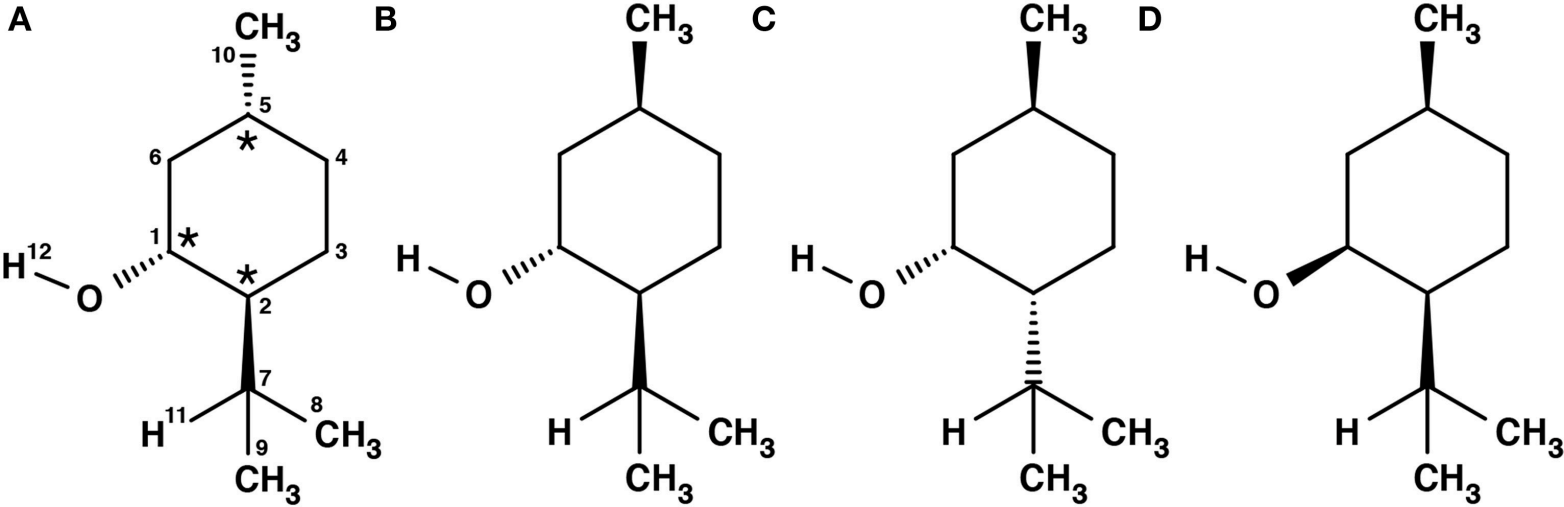
**Schemes of the structures of the (-)-enantiomers of the different stereoisomers of menthol: (A) (-)-menthol, (B) (-)-isomenthol, (C) (-)-neomenthol, (D) (-)-neoisomenthol**. In our sample, (-)-menthol (1R, 2S, 5R) is expected to be the main component. The three stereogenic centers are marked with stars in **(A)**.

Menthol is a monoterpene alcohol with several properties that make it a chemically and biologically interesting system. It is well-known for its minty smell and used as a flavoring agent in many different applications. Menthol is also famous for its cooling sensation when applied to skin or mucous membranes (Bautista et al., 2007). This property is exploited in its widespread use as a cooling enhancing additive in cigarettes, cough medicines, and antipruritics (Eccles, 1994; Kamatou et al., 2013). The three chiral centers of menthol result in eight different stereoisomers, of which the (-)-enantiomers are depicted in Figure 1. The stereoisomers, (-)-menthol (1R, 2S, 5R), and (-)-neomenthol (1R, 2R, 5S), are naturally occurring in the essential oil of Mentha x piperita L. (peppermint) with (-)-menthol as its major constituent and (-)-neomenthol as an impurity. The *R* (lat. rectus) or *S* (lat. sinister) are labels that specify the local configuration at each chiral center according to the Cahn, Ingold and Prelog nomenclature (Cahn et al., 1966).

**Figure 2:**
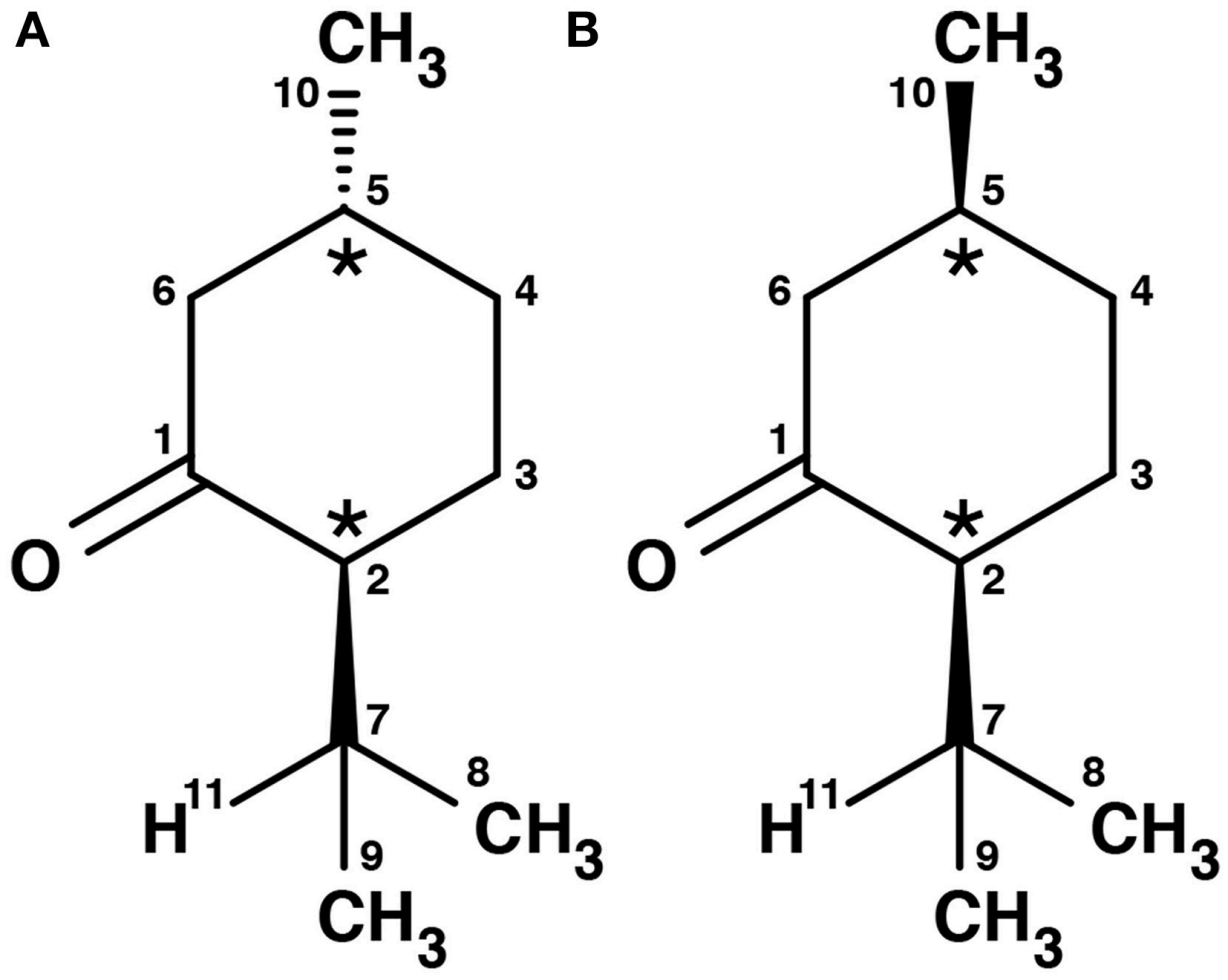
**Schemes of the structures of the (-)-enantiomers of (A) (-)-menthone, (B) (-)-isomenthone**. The two stereogenic centers are marked with stars.

Menthone is the ketone analog of menthol. It bears two chiral centers and hence four different stereoisomers. The (-)-enantiomers of menthone are shown in Figure 2. Menthone has a similar minty smell as menthol and also occurs naturally in peppermint oil. As with menthol, menthone is used as an odorant and within asymmetric synthesis (Harada et al., 1992). Both menthol and menthone also show potential as vehicles for transdermal drug delivery (Zhao et al., 2001; Brain et al., 2006; Kamatou et al., 2013).

Both molecules are of interest for spectroscopic studies due to their chiral properties. Comprising two stereogenic centers while maintaining a rather simple structure, menthone was used for demonstrating an enantiomeric excess determination technique (Shubert et al., 2014). Menthol served as prototype molecule in a chirality recognition study (Albrecht et al., 2010). The structure of menthol has been investigated before using electron diffraction, NMR, IR spectroscopy, and quantum chemical calculations (Egawa et al., 2003; Härtner and Reinscheid, 2008; Albrecht et al., 2010; Avilés Moreno et al., 2013). For menthone, only NMR and computational model data exist (Smith and Amezcua, 1998).

The conformational space of menthol is more complex than that of menthone due to the additional flexibility of the hydroxyl group. Menthol was extensively studied by Albrecht et al. (2010) and more recently by Moreno et al. in a combined IR and VCD spectroscopy study (Avilés Moreno et al., 2013). Herein, we build upon and relate our findings from microwave spectroscopy to their results. Moreover, we focus on the elucidation of the potential energy surface of menthone and its diastereomer isomenthone. Menthone and menthol share similar structural characteristics involving the lowest energy cyclohexane ‘chair’ configuration, while the low-energy conformational landscape of isomenthone retains several cyclohexane conformations.

Broadband microwave spectroscopy enables the simultaneous measurement of the rotational spectra of multiple conformations, providing a wide swath of the spectrum within the bandwidth of the instrument in a single measurement. This broad acquisition is beneficial for the assignment of the individual transitions within a spectrum because recurring patterns can be identified quickly and linked to a predicted spectrum. However, the presence of several species in the rotational spectrum can impede a straightforward assignment. Therefore, we developed and used a computer program for the analysis of broadband microwave spectra and applied it to the mixture of menthone isomers (four different species). The program identifies and fits the different species within a spectrum using calculated ab initio rotational constants as initial input values.

## 2. Experimental and computational methods

Menthone [2-isopropyl-5-methylcyclohexanone, 97% purity, mixture of isomers, i.e., potentially containing all four stereoisomers (±)-menthone and (±)-isomenthone] and menthol (2-isopropyl-5-methylcyclohexanol, 99% purity) were purchased from Sigma-Aldrich Chemie GmbH, Taufkirchen, Germany and were used without further purification. For menthol no further information was provided about the stereoisomeric composition. Due to its synthetic origin, the stereoisomer (-)-menthol is expected to be the main component. Menthol is a white, flaky, and sticky hydrophilic crystalline solid with a stated melting point of 34–36°C and a boiling point of 216°C at standard conditions. The mixture of menthone isomers is a yellowish liquid with a stated boiling point of 85°C. The schemes of the chemical structures of (-)-menthone, (-)-isomenthone and (-)-menthol are depicted in Figures 1, 2.

All rotational spectroscopy measurements were performed with the Hamburg COMPACT spectrometer which has been detailed elsewhere (Schmitz et al., 2012) and thus only a brief description is given here. The molecules were seeded into a supersonic expansion using a pulsed nozzle (Parker General Valve, Series 9) operating at 2 Hz. The sample holders were placed directly prior to the nozzle and heated to about 112°C for both menthol and menthone. This temperature gave the best signal for both molecular samples. A constant flow of neon at a stagnation pressure of 3.0 bar transported the molecules to the nozzle. After supersonic expansion into vacuum, the ensemble of molecules was polarized with a 1 μs chirp spanning 2 → 8.5 GHz. The chirp was generated with an arbitrary waveform generator (AWG), amplified to 300 W with a traveling wave tube amplifier, and transmitted into the vacuum chamber with a horn antenna. Following excitation, the free induction decay (FID) of the macroscopic ensemble of polarized molecules was recorded. For each spectrum, 50 μ s of the FID were recorded at a resolution of 10 ps, yielding a frequency resolution of 20 kHz in the Fourier transform (FT) and hence the rotational spectra obtained. For menthol and menthone, 444 000 FIDs and 128 000 FIDs, respectively, were coadded for each spectrum. The resulting signal to noise ratio of (100:1) for the strongest transitions was sufficient to identify the parent isotopologue species of the different conformers. After performing the Fourier transformation, residual background lines were removed.

Herein, the experimental results were compared with electronic structure calculations performed with the Gaussian 09 (Frisch et al., 2009) program suite. For menthol, in order to determine the preferred orientations of the hydroxyl group and the lowest energy orientation of the isopropyl group, we carried out relaxed energy scans of rotations for the hydroxyl and isopropyl groups with respect to the cyclohexane ring. We employed the Becke, three parameter, Lee-Yang-Parr (B3LYP) exchange-correlation functional with the 3-21G and the 6-311++G(d,p) basis sets for these potential energy surface (PES) scans. To further refine the structures and verify the energy ordering of the individual conformers, optimizations and harmonic frequency calculations were carried out on all minima using several different basis sets and methods: B3LYP/6-311++G(d,p), B3LYP/aug-cc-pVTZ, and MP2/6-311++G(d,p) (Møller– Plesset second order perturbation theory). Furthermore, the Minnesota functional M06-2X with the 6-311++G(d,p) basis set was used to optimize the structure of the conformers. The rotational constants and dipole moments agree well with the B3LYP and MP2 calculations, but the relative zero-point corrected energies differ by about 1 kJ/mol for some conformers. The results of the M06-2X calculations are compiled in the supplementary information, for which significant differences in the zero-point corrected energies are observed. In order to get an overview of the internal dynamics, the barrier to internal rotation for the different methyl tops was calculated at the B3LYP/6-311++G(d,p) level of theory.

## 3. Results and discussion

### 3.1. Menthol

**Figure 3:**
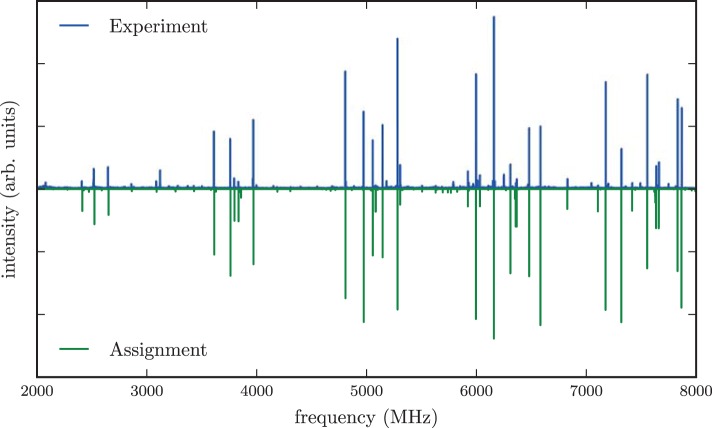
**Microwave spectrum of menthol in the region of 2–8 GHz**. The upper trace shows the experimental spectrum, the lower trace is a simulation based on fitted molecular parameters. The spectrum is dominated by *a*-type and *c*-type transitions. A rotational temperature of 0.5 K gives the best match between the simulated and the experimental intensities. A line splitting due to the internal rotation of any of the methyl groups is not observed in the spectrum.

The experimental spectrum of menthol is presented in the positive trace of Figure 3. The spectrum is typical for a prolate asymmetric rotor with μ_*a*_ as the largest dipole moment component. We assigned and fitted 32 *a*-type and 21 *c*-type lines, but no *b*-type lines were observed in the spectrum. The spectrum arises from only a single species, indicating that only one conformer of menthol is significantly populated in the supersonic expansion, in line with the findings of a recent FTIR study (Albrecht et al., 2010). Furthermore, it confirms that menthol is indeed the main stereoisomer in the purchased sample, since isomenthol, neomenthol and neoisomenthol all have different rotational constants and thus can be easily differentiated by their rotational spectra. For fitting the spectrum, we used Pickett's SPFIT/SPCAT program suite employing the *I*^*r*^ representation and the Watson A reduction (Pickett, 1991). Table 1 summarizes the results of the fit. We observed and assigned lines including rotational quantum numbers *J* = 1 to *J* = 7 and a maximum *K*_*a*_ of 4.

**Table 1:** **Experimentally obtained and calculated rotational constants and dipole moments for menthol**.

	**Experiment**	**Conformer EQ1ext**
*A* (MHz)	1779.79549(38)	1776/1769/1795
*B* (MHz)	692.62171(24)	686.1/683.0/699.7
*C* (MHz)	573.34542(27)	569.8/567.6/574.5
Δ_*J*_ (kHz)	0.0166(31)	–
μ_*a*_ (D)	–	1.3/1.3/1.4
μ_*b*_ (D)	–	–0.1/–0.1/–0.1
μ_*c*_ (D)	–	0.8/0.9/0.8
*N*°(a/b/c)	53 (32/0/21)	–
σ (kHz)	5.5	–

The errors given here are standard errors. N°(a/b/c) gives the number of transitions included in the fit according to its type, and σ is the standard deviation of the fit. The computational results for conformer EQ1ext were obtained using three different combinations of calculation methods and basis sets and they are given in the following order: B3LYP/aug-cc-pVTZ / B3LYP/6-311++G(d,p) / MP2/6-311++G(d,p).

The conformational flexibility of menthol originates from the cyclohexane configuration and the orientations of the hydroxyl and isopropyl groups. Rotations of the different methyl groups do not contribute to the conformational space because they have *C*_3*v*_ local symmetry for which rotation gives rise to three equivalent minima. However, they could lead to characteristic tunneling splittings if the corresponding barriers are sufficiently low.

**Figure 4:**
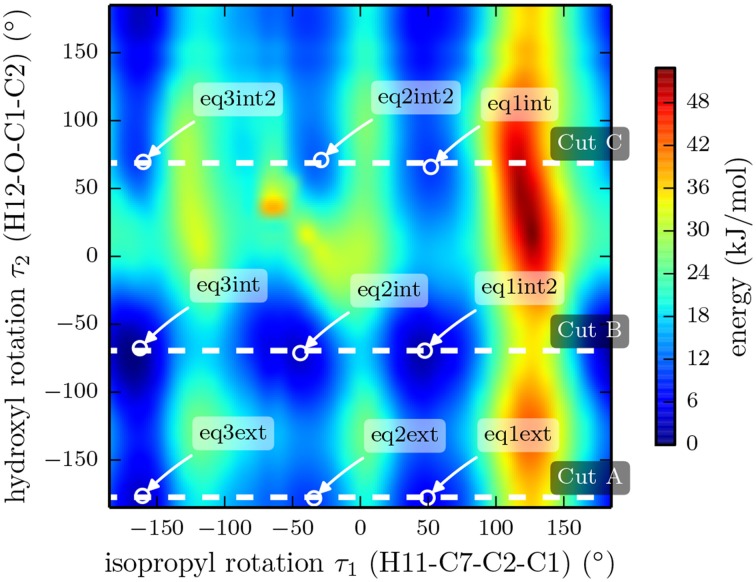
**Plot of the potential energy landscape of (-)-menthol as a function of the isopropyl and the hydroxyl dihedral angles**. The data was extracted from a two-dimensional, relaxed potential energy scan using the B3LYP/3-21G functional. The dashed lines mark cuts through the 3D surface, which were evaluated using a higher level of theory (B3LYP/6-311++G(d,p)). The results of these three scans are depicted in Figure 5.

It was shown by NMR spectroscopy, gas phase electron diffraction, and several quantum chemical computation studies that the chair conformation of cyclohexane is preferred in menthol (Egawa et al., 2003; Härtner and Reinscheid, 2008). In order to get an overview of the potential energy surface, we performed a relaxed two dimensional scan at the B3LYP/3-21G level of theory by rotating the isopropyl and hydroxyl groups with all three substituents in equatorial positions. The rotation of the hydroxyl group with respect to the ring occurs around the dihedral angle τ_2_ (H12-O-C1-C2), while the isopropyl orientation is described with the dihedral angle τ_1_ (H11-C7-C2-C1) (compare Figure 1). The resulting PES is shown in Figure 4 and reveals the existence of various stable rotamers. In this figure we mark the nine lowest energy rotamers using the labeling scheme introduced by Avilés Moreno et al. (2013).

**Figure 5:**
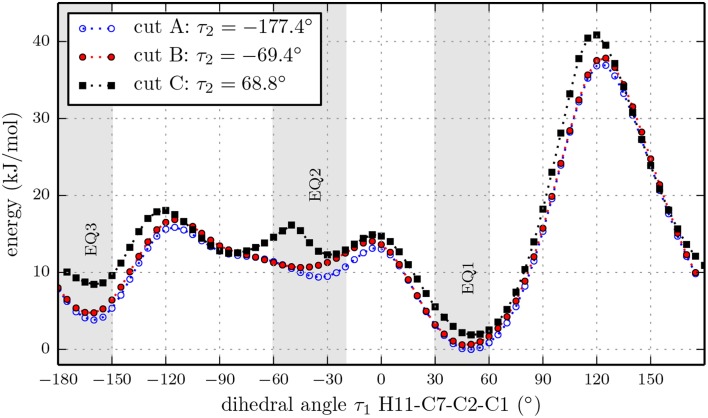
**Relaxed potential energy scans for the rotation of the isopropyl group of (-)-menthol with fixed orientations of the hydroxyl group at dihedral angles τ_2_ = [−177.4,−69.4,68.8]° (H12-O-C1-C2) according to the marks in Figure 4, at the B3LYP/6-311++G(d,p) level of theory**. All three traces have the same overall features, while cut A contains the lowest minima. The double minimum structure of cut C around EQ2 might be an artifact due to fixing dihedral angle τ_2_.

We also performed three relaxed one-dimensional scans of the rotation of the isopropyl group with a larger basis set and the hydroxyl dihedral angle τ_2_ fixed at values of −177.4°, −69.4° and 68.8°. These three cuts through the three-dimensional potential energy surface are indicated by the white dashed lines in Figure 4 and were carried out using the B3LYP functional and the 6-311++G(d,p) basis set. The results are presented in Figure 5. The slices go through the nine lowest energy structures and give a better understanding of the energy ordering. At the minima positions EQ1 and EQ3, the isopropyl group is out of plane with respect to the cyclohexane ring. At the minima positions EQ2, the isopropyl group is in plane but the hydrogen H11 is opposite to the hydrogen bonded to C2 (see Figure 1). These structures are much higher in energy than those at the minima positions EQ1 and EQ3. The orientation of the hydroxyl group at an angle of τ_2_ = −177.4° gives the lowest energy trace.

This relaxed potential energy scan revealed the energy ordering of the different conformers, and we chose the five lowest energy conformers (EQ1ext, EQ1int2, EQ1int, EQ3ext and EQ3int) below 6 kJ/mol for further analysis at the B3LYP/aug-cc-pVTZ, B3LYP/6-311++G(d,p), and MP2/6-311++G(d,p) levels of theory. The zero-point corrected energies, rotational constants, and dipole moments from these calculations are listed in Table 2. EQ1ext is the lowest energy conformer at all three levels of theory, in agreement with the gas phase electron diffraction data by Egawa et al. (2003). We also carried out relaxed potential energy scans on conformer EQ1ext for the rotation of the methyl groups. The corresponding barrier heights are around 12 *kJ*/mol, too high to affect the observed rotational spectrum and in agreement with our observations.

**Table 2:** **Calculated relative zero-point corrected energies, rotational constants, dipole moments, and torsional angles for the five lowest energy conformers of menthol**.

**Conformer**	**EQ1ext**	**EQ1int2**	**EQ1int**	**EQ3ext**	**EQ3int**
Δ*E* (kJ/mol)	0.0/0.0/0.0	1.0/0.9/1.4	1.9/2.0/2.1	4.5/4.2/3.3	5.3/5.0/5.0
*A* (MHz)	1776/1769/1795	1760/1753/1774	1762/1755/1777	1954/1948/1975	1939/1932/1960
*B* (MHz)	686.1/683.0/699.7	685.0/682.1/698.0	687.2/684.6/699.1	668.2/665.7/674.7	668.4/665.9/674.1
*C* (MHz)	569.8/567.6/574.5	589.0/566.8/573.5	568.6/566.1/573.1	575.6/573.6/582.7	574.4/572.2/581.5
τ_1_(°)^a^	49.1/49.5/58.3	47.4/47.6/56.1	51.0/52.0/58.3	–160.5/–160.7/–158.7	–162.1/–162.5/–159.5
τ_2_(°)^a^	–176.4/–177.8/–173.2	–69.7/–69.4/–66.6	66.0/65.9/62.3	–175.3/–176.6/–172.3	–67.1/–67.6/–67.7
μ_*a*_ (D)^a^	1.3/1.3/1.4	0.8/0.9/0.8	–0.8/–0.9/–0.9	1.5/1.5/1.6	1.0/1.1/1.1
μ_*b*_ (D)^a^	–0.1/–0.1/–0.1	–1.1/–1.2/–1.3	–1.2/–1.2/–1.3	–0.1/–0.1/–0.03	–1.0/–1.1/–1.0
μ_*c*_ (D)^a^	0.8/0.9/0.8	–0.7/–0.7/–0.7	0.9/0.9/0.9	0.8/0.9/0.8	–0.8/–0.9/–0.9
	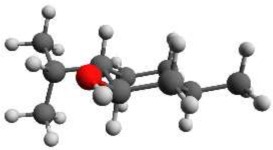	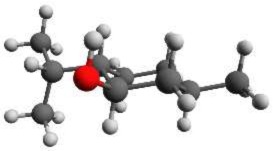	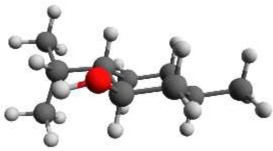	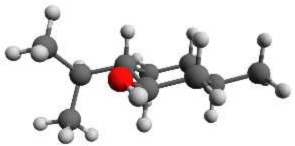	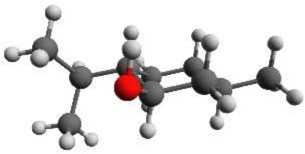

We use the labeling scheme introduced by (Avilés Moreno et al., 2013). The results were obtained using three different combinations of calculation methods and basis sets and they are given in the following order: B3LYP/aug-cc-pVTZ / B3LYP/6-311++G(d,p) / MP2/6-311++G(d,p).

a Dihedral angles and dipole moment components are given for (-)-menthol.

The similarity of the rotational constants for the calculated structures EQ1ext, EQ1int2, and EQ1int complicate a straightforward assignment of the fitted constants of the single observed menthol conformation (Table 1) to one of the three lowest energy conformers compiled in Table 2. However, the energy ordering of the conformers, which is the same for all calculation methods and basis sets employed, suggests that the lowest energy conformer EQ1ext is the observed one. This observation is supported by the calculated dipole moment components of the menthol conformers. Conformer EQ1ext has a very weak dipole moment component along the *b*-axis and no *b*-type lines were observed in the spectrum. However, for the higher energy conformers, EQ1int2 and EQ1int, μ_*b*_ is predicted to be the strongest dipole moment component. For the conformers EQ3ext and EQ3int, the calculated *A*-rotational constants differ by 200 MHz from the experimentally determined one. Hence, we assigned the observed menthol conformation to the lowest energy conformer, EQ1ext. It should be noted that the deviations of the rotational constants for the four different theoretical levels employed here are of the same order of magnitude as the deviations between the three EQ1 conformers (i.e., EQ1ext, EQ1int2, and EQ1int). The predictions of the dipole-moment components, however, seem to be significantly more reliable and allow for the structural assignment of the observed menthol conformer, as stated above.

The observation of only a single menthol conformation can be explained by the potential energy surface. The three lowest energy conformers (EQ1ext, EQ1int, EQ1int2) differ in the rotation of the hydroxyl group. The barrier of this rotation between each conformer is around kJ/mol (B3LYP/6-311++G(d,p)). As this barrier height is small, it is expected that the supersonic expansion cools conformers EQ1int and EQ1int2 into EQ1ext. The sets of conformers EQ2 and EQ3 are much higher in energy by 5−10^kJ^/mol and, even though the populations in EQ3 might be frozen during supersonic expansion due to the higher isomerization barriers to EQ1 and EQ2, their populations remain too low to affect the spectrum. The observation of the conformer EQ1ext agrees with the gas electron diffraction study of Egawa et al. (2003). In comparison to the conformers EQ1int and EQ1int2, conformer EQ1ext might be stabilized by a weak non-conventional hydrogen bond between H11 and the hydroxyl O (Albrecht et al., 2010). The signal to noise ratio of approximately 100:1 for the most intense lines did not permit the observation of lines originating from ^13^C- or ^18^O-isotopologs of menthol to enable more advanced structure determination.

### 3.2. Menthone and isomenthone

**Figure 6:**
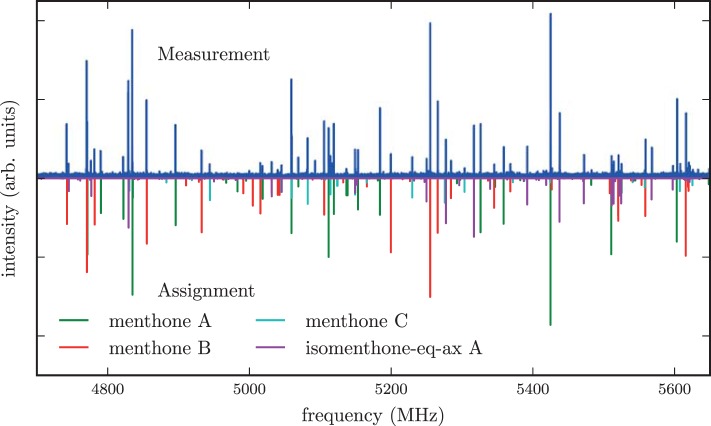
**The upper trace is the spectrum of the mixture of the stereoisomers of menthone and isomenthone in the region of 4700–5650 MHz while the lower traces are simulations based on fitted molecular parameters**. Three different conformers of menthone and one conformer of isomenthone were identified in the spectrum and successfully assigned. A rotational temperature of 1.5 K for all four species gives the best match between the simulated and the experimental intensities. A few residual lines with SNR ratios of about 3:1 remain unassigned, which might originate from contaminants, clusters or instrument noise. Line splittings due to internal rotation of one of the methyl groups of menthone or isomenthone were not observed.

The spectrum of the mixture of menthone isomers is presented in the frequency range from 4700 to 5650 MHz in the positive trace of Figure 6. The high number and density of lines of molecular origin and various background lines complicated the assignment of the individual lines to the particular species. Therefore, a computer-aided assignment routine was developed to facilitate the assignment process. This routine is described in Section 3.3. It was possible to assign most of the lines to four different species. For all species, most of the assigned transitions are *b*-type, with fewer *a*-type transitions and even fewer *c*-type transitions. This observation suggests that for all four species μ_*b*_ > μ_*a*_ > μ_*c*_ applies. The final fits were executed employing the SPFIT/SPCAT suite of programs using the *I*^*r*^ representation and the Watson A reduction. The results are compiled in Table 3.

**Table 3:** **Experimentally determined rotational constants and quartic distortion constants of menthone and isomenthone**.

	**Menthone A**	**Menthone B**	**Menthone C**	**Isomenthone eq-ax A**
*A* (MHz)	1953.43379 (43)	2021.98637 (36)	2109.38469 (65)	1535.27577 (48)
*B* (MHz)	694.51551 (19)	693.53686 (16)	681.13604 (27)	812.92526 (33)
*C* (MHz)	586.57758 (19)	562.13636 (16)	598.12413 (25)	671.43466 (33)
Δ_*J*_ (kHz)	0.0106 (18)	0.0109 (18)	0.0183 (37)	0.0651 (53)
Δ_*JK*_ (kHz)	0.0234 (36)	0.0473 (50)	0.097 (11)	−0.1844 (60)
Δ_*K*_ (kHz)	–	–	–	0.535 (20)
*N*° (a/b/c)	92 (26/66/0)	112 (31/62/19)	57 (21/32/4)	76 (6/67/7)
σ (kHz)	7.4	6.0	5.8	7.5

The errors given here are standard errors. *N*°(a/b/c) is the number of lines included in the fit distributed for *a*-, *b*-, and *c*-type transitions, and σ is the standard deviation of the fit.

**Figure 7:**
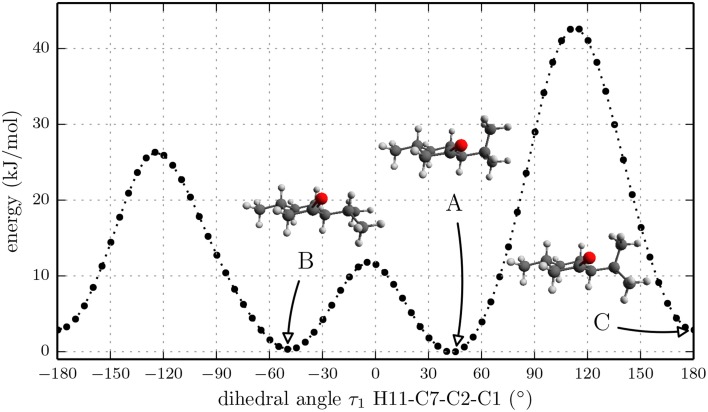
**Scan of the potential energy surface calculated along the dihedral angle τ_1_ (H11– C7– C2– C1) according to the labeling in Figure 2 at the B3LYP/6-311++G(d,p) level of theory for (-)-menthone**. The position of each minimum conformation (A, B, C) is indicated in the plot and illustrated with the corresponding structure.

According to NMR measurements and quantum chemical calculations by Smith et al., the cyclohexane ring in menthone is in the chair configuration and both substituents, the isopropyl and the methyl groups, are in equatorial positions (Smith and Amezcua, 1998). Hence the degrees of freedom of menthone are limited to the rotations of the isopropyl and methyl groups. We calculated the relaxed potential energy surface for the rotation of the isopropyl group [dihedral angle τ_1_ (H11-C7-C2-C1)] at the B3LYP/6-311++G(d,p) level of theory. The result is displayed in Figure 7 and exhibits three different minima at orientations similar to those of (-)-menthol. These three structures were optimized at the B3LYP/aug-cc-pVTZ, B3LYP/6-311++G(d,p), and MP2/6-311++G(d,p) levels of theory. The results are summarized in Table 4. The zero-point corrected energies of the three menthone rotamers are within 3 *kJ*/mol (B3LYP/aug-cc-pVTZ calculation) of each other.

**Table 4:** **Calculated relative zero-point corrected energies, Boltzmann populations, rotational constants, dipole moments, and torsional angles for the three lowest energy conformers of menthone**. The results were obtained using three different combinations of theoretical methods and basis sets and they are stated in this order: B3LYP/aug-cc-pVTZ / B3LYP/6-311++G(d,p) / MP2/6-311++G(d,p).

	**Menthone A**	**Menthone B**	**Menthone C**
	**B3LYP^b^/B3LYP^c^/MP2^c^**	**B3LYP^b^/B3LYP^b^/**MP2**^c^**	**B3LYP^b^/B3LYP^c^/**MP2**^c^**
Δ*E* (kJ/mol)	0.0/0.0/0.84	0.19/0.29/0.0	3.0/2.9/3.9
Population (%)^a^	42.9/43.1/48.4	40.4/39.4/37.2	16.7/17.5/14.5
*A* (MHz)	1950/1941/1965	2017/2009/2029	2105/2096/2117
*B* (MHz)	688.7/686.1/694.1	689.3/686.4/695.2	675.7/673.1/684.5
*C* (MHz)	582.4/579.9/590.0	558.7/556.2/566.3	593.3/591.0/601.7
μ_*a*_ (D)^d^	1.3/1.3/1.2	–1.3/–1.3/–1.2	1.8/1.8/–1.6
μ_*b*_ (D)^d^	–2.6/–2.7/–2.5	–2.4/–2.5/–2.2	–2.2/–2.3/2.0
μ_*c*_ (D)^d^	–0.5/–0.5/–0.5	1.1/1.2/1.2	–1.1/–1.1/–1.2
τ_1_(°)^d^	42.6/42.9/41.9	–48.7/–48.8/–49.3	–179.8/–179.8/176.7
	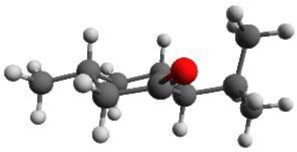	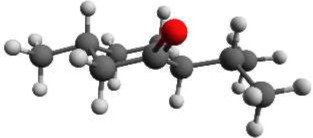	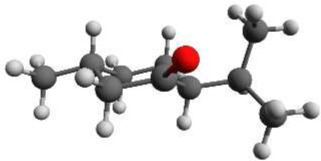

a Boltzmann distribution calculated at the sample reservoir temperature of 386 K.

b aug-cc-pVTZ basis set.

c 6-311++G(d,p) basis set.

d Dipole moment components and dihedral angle are given for the (-)-enantiomer.

While all theoretical methods employed show comparable trends for both the rotational constants and the dipole-moment components, we find some differences in the relative zero-point corrected energies for the individual conformers. For example, using MP2/6-311++G(d,p) calculations, menthone B is predicted to be the lowest-energy conformer by 0.8 *kJ*/mol, while for all other approaches, menthone A is predicted to be the lowest-energy species (Table 4). An explanation of these results is not straightforward. The two menthone conformers A and B differ in the orientation of the isopropyl group with respect to the cyclohexanone ring. The minimum orientations are the result of an interplay between intramolecular interactions, such as van der Waals and dispersion interactions, potential hydrogen bonding to the ketone oxygen, or steric hindrance (repulsion of electron clouds). These different contributions to the final energy are considered differently in the various theoretical approaches (for example, the B3LYP approach does not account for dispersion that is empirically treated in the M06-2X approach and explicitly considered in MP2 calculations).

Thus, although we can unambiguously assign structures to the three experimentally determined menthone conformers (Table 3), we cannot determine their precise energy ordering for this particular case.

For isomenthone (cis-configuration) the situation is different. Isomenthone may exist with the isopropyl and the methyl group substituent either equatorial or axial, which in turn influences the cyclohexane configuration. Intuitively, three different starting structures are feasible: the methyl group oriented equatorial and the isopropyl group axial to the cyclohexane ring (isomenthone eq-ax), the methyl group oriented axial and the isopropyl group equatorial to the cyclohexane ring (isomenthone ax-eq) and both substituents oriented equatorial to the ring (isomenthone eq-eq). The diaxial configuration always relaxes to one of the other conformers during the structural optimizations. It is destabilized by the high steric strain between the two substituents.

**Figure 8:**
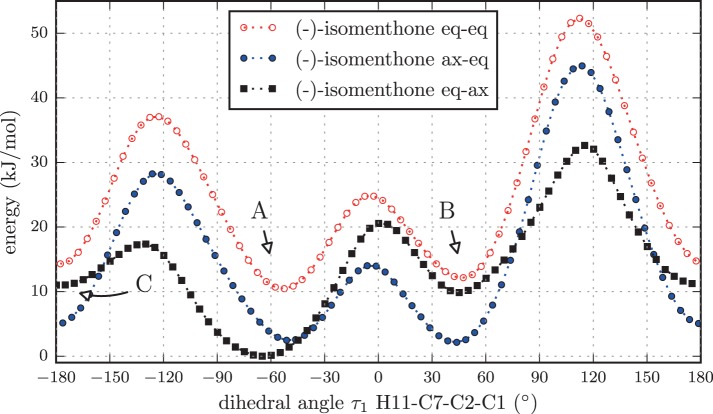
**Scan of the potential energy surface calculated along the dihedral angle τ_1_ (H11– C7– C2– C1) according to the labeling in Figure 2 at the B3LYP/6-311++G(d,p) level of theory for (-)-isomenthone eq-eq, (-)-isomenthone ax-eq and (-)-isomenthone eq-ax**. The labels A–C are pointing to the corresponding minima. Only the conformation isomenthone eq-ax A was observed in the microwave spectrum.

Again we performed a relaxed potential energy scan by rotating the isopropyl group around the dihedral angle τ_1_ for the three different starting structures (-)-isomenthone eq-ax, (-)-isomenthone ax-eq and (-)-isomenthone eq-eq. Figure 8 shows the results for these relaxed potential energy scans. The global minimum was found for (-)-isomenthone eq-ax at about τ_1_ = −60°. Therefore, the set of conformers with τ_1_ being close to −60° is labeled as A. The set of minimum structures at τ_1_ ≈ −45° is labeled as B, and the set at τ_1_ ≈ −180° is labeled as C. For all nine minimum structures, optimizations and frequency calculations were carried out using the B3LYP/aug-cc-pVTZ, B3LYP/6-311++G(d,p), and MP2/6-311++G(d,p) levels of theory. The results of these calculations for the three lowest energy structures (isomenthone eq-ax A, isomenthone ax-eq A, and isomenthone ax-eq B) are listed in Table 5. The dipole moment components and the dihedral angle of the isopropyl group are given for the (-)-enantiomers.

**Table 5:** **Calculated relative zero-point corrected energies, Boltzmann populations, rotational constants, dipole moments, and torsional angles for the three lowest energy conformers of isomenthone**. The results were obtained using three different combinations of theoretical methods and basis sets and they are stated in this order: B3LYP/aug-cc-pVTZ / B3LYP/6-311++G(d,p) / MP2/6-311++G(d,p).

	**Isomenthone eq-ax A**	**Isomenthone ax-eq A**	**Isomenthone ax-eq B**
	**B3LYP^b^/B3LYP^c^/MP2^c^**	**B3LYP^b^/B3LYP^c^/MP2^c^**	**B3LYP^b^/B3LYP^c^/MP2^c^**
Δ*E* (kJ/mol)	0.0/0.0/0.0	2.7/2.5/3.7	2.7/2.5/4.4
Population (%)^a^	44.6/43.2/58.4	19.5/20.0/18.6	19.1/20.0/14.9
*A* (MHz)	1538/1528/1516	1848/1839/1849	1731/1721/1741
*B* (MHz)	804.5/802.2/824.6	765.3/762.6/781.4	771.0/768.8/788.6
*C* (MHz)	662.3/660.2/680.6	624.8/622.5/641.0	674.1/672.0/693.4
μ_*a*_ (D)^d^	–0.7/–0.7/–0.6	–1.1/–1.1/–0.9	1.1/1.1/0.9
μ_*b*_ (D)^d^	–3.1/–3.1/–2.9	2.5/2.4/2.2	–2.7/–2.7/–2.5
μ_*c*_ (D)^d^	–1.0/–1.0/–1.0	–1.4/–1.4/–1.4	–0.7/–0.8/–0.7
τ_1_(°)^d^	–64.5/–64.1/–64.0	–49.4/–49.4/–49.2	42.6/42.9/42.0
	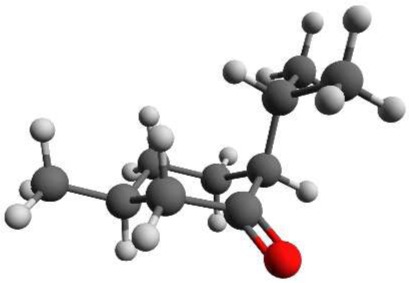	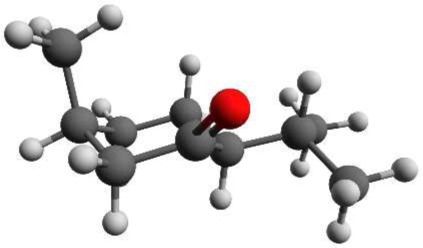	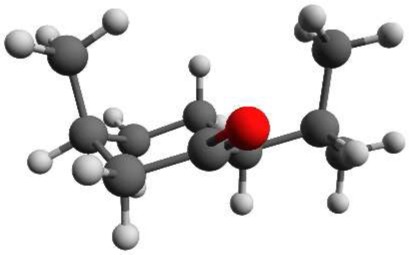

a Boltzmann distribution calculated at the sample reservoir temperature of 386 K.

b aug-cc-pVTZ basis set.

c 6-311++G(d,p) basis set.

d Dipole moment components and dihedral angle are given for the (-)-enantiomer.

Considering only the three lowest energy conformers for menthone and isomenthone (Tables 4, 5) the assignment of the experimentally determined species to the calculated structures is straightforward. At least one of the experimentally determined rotational constants differs significantly from the calculated ones for isomenthone ax-eq A and isomenthone ax-eq B, so that we assigned three of the observed species to menthone A, menthone B, and menthone C and the fourth one to isomenthone eq-ax A. In addition to the rotational constants, we included the distortion constants Δ_*J*_ and Δ_*JK*_ for all conformers. Inclusion of the constant Δ_*K*_ only improved the fitting results of conformer isomenthone eq-ax A. The axially aligned isopropyl group of isomenthone eq-ax seems to lead to a higher sensitivity regarding centrifugal distortion.

For the three lowest-energy conformers of isomenthone, the different theoretical approaches agree well with each other and the lowest-energy form eq-ax A matches the experimentally determined values for the rotational constants. The calculated relative zero-point corrected energies are also similar when comparing the B3LYP- and MP2-based approaches. However, for calculations employing the M06-2X density functional, significantly different energies are predicted (Supplementary Tables 8–10). While we again find a good agreement for the rotational constants and dipole-moment components, the three low-energy conformers given in Table 5 are predicted to be nearly isoenergetic using the M06-2X functional, for which we do not have an explanation and which is also not predicted by the other approaches.

The cyclohexane ring in all three observed menthone conformers is in a chair configuration and both substituents in equatorial orientations. Such a structural arrangement is expected due to steric effects. Hence, the conformational space of menthone is governed by the isopropyl rotation. The barriers separating the different menthone conformers are between 12 kJ/mol and 40 kJ/mol and should be high enough to freeze the population to that at the temperature of the sample reservoir during the cooling process of the supersonic expansion. The situation is different for isomenthone. Both substituents are arranged cis to the cyclohexane ring. Hence, the equatorial orientation of both substituents forces the cyclohexane ring into the boat configuration, which is higher in energy compared to the chair configuration for unsubstituted cyclohexane. The diaxial orientation of the substituents is prevented by steric hindrance. Consequently, in the lowest energy conformers of isomenthone one substituent needs to be oriented equatorially and the other one axially. We think that due to the anomeric effect the lowest energy conformer isomenthone eq-ax A having the isopropyl group oriented axially is stabilized. It is surprising that only one conformer of isomenthone was observed. Using the argument that the conformers are frozen out due to the barrier height, we would expect more conformers of the ax-eq kind. This absence might be explained by the fact that the fraction of isomenthone in the mixture is only 30% (analysis by Sigmar-Aldrich), which is confirmed by weaker intensities of isomenthone (SNR of approximately 50:1 for the strongest transitions) compared to menthone A (SNR of approximately 100:1 for the strongest transitions). Thus, the density of the additional conformations in the expansion is likely too low to allow them to be observed with the signal-to-noise ratio of the spectrum we present here, i.e., with only 128000 single acquisitions coadded.

### 3.3. Computer aided assignment routine

The assignment of the spectrum from the menthone/isomenthone mixture was complicated by the overlap of different *J*-transitions that impeded a straightforward analysis. The presence of background lines led to further complications. Therefore, we developed a computer program to facilitate the analysis. Earlier approaches to automate spectral assignment include a computer program based on a genetic algorithm to fit the details of a rotationally resolved UV spectrum and subsequently extract the molecular parameters of multiple mixture components (Meerts and Schmitt, 2006). This approach was implemented with a set of molecular parameters representing a chromosome and thus the molecular parameters of all molecules present in the mixture must be within the chromosome. The fitness of a solution was determined by a direct comparison of the measured and predicted spectrum.

Recently, Seifert and coworkers introduced another approach for an automated assignment routine called Autofit (Seifert et al., 2015). Their method is based on the assignment of three (linearly independent) lines, representing the minimum number of lines for fitting an asymmetric rotor spectrum including only rotational constants. Within a predefined bandwidth, the program determines potential peaks for each transition, fits all possible combinations of the assignments, and uses the error of the fit to evaluate the solutions. The performance of this program was tested on multiple silicon-containing species and was capable of identifying not only the parent species, but also its carbon and silicon isotopologues (Seifert et al., 2013).

Here we present another approach for an automatic assignment and fitting procedure with the aim of determining the rotational constants to a high precision. In contrast to the Autofit program, which assigns and fits three different peaks directly, we start by scanning the parameter space of rotational constants in order to evaluate the goodness of different parameter sets. Only the best parameter sets are used in all subsequent steps, which reduces the computation time drastically. In a second step, four transitions are assigned automatically depending primarily on the frequency and intensity overlap between the measured and predicted spectra. Three transitions are the absolute minimum to fit three rotational constants, but only when the effect of the rotational constants on the assigned transitions is sufficiently significant. Therefore, we choose to use four transitions to stabilize the fit and reduce the number of diverging results. Diverging and unphysical results are directly excluded and the quality of the fit is assessed by a comparison of the measured and predicted spectra. In the following we explain the algorithm in detail using the example of isomenthone eq-ax A and highlight the advantages and potential pitfalls.

#### Step 1: definition and filling of the scan volume

As previously mentioned, we work in a three-dimensional parameter space of rotational constants with their values along the three axes. In the graphical presentation, the samples are defined by (A, B, C)-triples plus a color-code describing their fitness. Two parameters are crucial to the performance of the routine: the volume size and the sample density. The volume should contain the rotational constants of the measured species. Hence the choice of the scan volume depends on the accuracy of the first guess, typically coming from quantum chemical calculations. As stated above, all theoretical approaches employed here give comparable rotational constants, which is a promising prerequisite for the computer-assisted fitting procedure.

**Figure 9:**
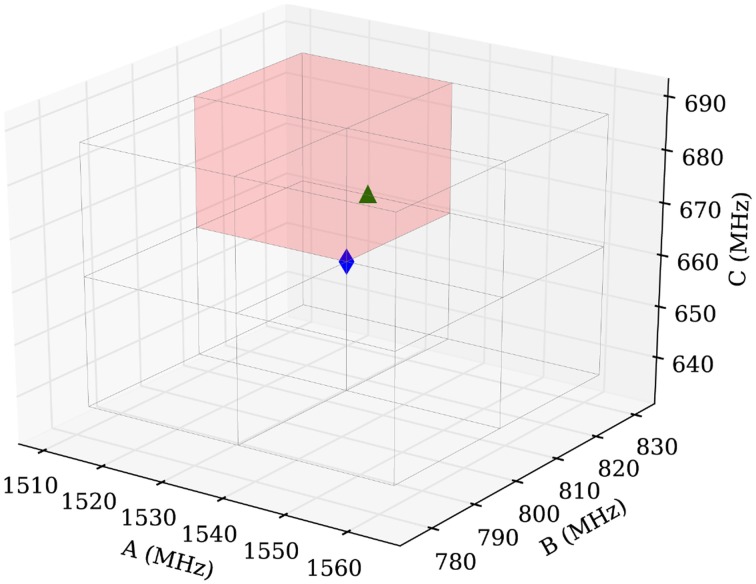
**Scheme of the sampling volume of the rotational constants for the computer aided assignment routine**. The cubic sampling volume is divided into eight subcubes and the routine is carried out consecutively in each of the subcubes. Afterwards the results of the individual subcubes are compared. The blue diamond in the center indicates the position of the calculated rotational constants and the green triangle indicates the position of the experimentally obtained value for isomenthone eq-ax A. The red shaded area is used to display the results in Figure 10.

A disadvantage to increasing the scan volume is the increased probability that the routine converges to an artificial local minimum. To avoid this scenario, we divide the initial volume into smaller parts and run the routine in each of them and compare the results afterwards. In practice, we choose a cubic volume with 50 MHz edge length centered at the calculated values (blue diamond in Figure 9) and subdivide the volume equally into eight subcubes. This procedure is illustrated in Figure 9, where the blue diamond indicates the position of the calculated values and the green triangle is positioned on the manually fitted values.

The choice of the sample density is essential for the successful assignment, but also the most important factor on the computation time. The sample density is correlated with the density of transitions in the spectrum. A spectrum with a high density of transitions requires a higher sample density for successful performance. We sample a subcube with a set of 2000 randomly distributed points.

A prediction is made for each point and an intensity cutoff is applied for both the experimental (*N* = 561) and the predicted data. Then both sets of transitions (experiment and prediction) are compared and an assignment is noted for each experimental peak within a predefined bandwidth of a predicted transition. If multiple experimental peaks are found within this bandwidth, only the closest peak is used for the assignment. The bandwidth for the assignment is closely related to the sample density. A higher sample density allows a smaller bandwidth, a low sample density requires a larger bandwidth. In our case, a bandwidth of 10 MHz gives good results in all our tests.

Finally, the mean-square value of the assignments is calculated and used to judge the fitness of the prediction. The samples are ranked according to their fitness and only the most promising candidates (in our case, the best 10%) are selected for the subsequent, computationally more demanding fitting procedure.

#### Step 2: assignment and fitting of the samples within a subcube

In this step, the best results of step one are used as starting point for the fitting procedure using SPFIT. Because only four transitions are used to fit the three rotational constants, the quality of these assignments is essential. Since the correct solution can only be obtained if the four transitions are assigned correctly in at least one trial, care must be taken in choosing the appropriate four transitions for fitting. As a selection criteria for choosing these transitions, we use the predicted intensities of the assigned transitions. To avoid errors in the predicted intensities from the use of incorrect simulation temperatures or dipole moments, we choose the ten transitions with the highest predicted intensities. From these ten assignments, we choose four at random to increase the diversity of transitions. Sometimes multiple experimental transitions can be assigned to a predicted line. In such a case, as many as four consecutive fits are performed assigning the four most intense experimental peaks to a predicted line.

**Figure 10:**
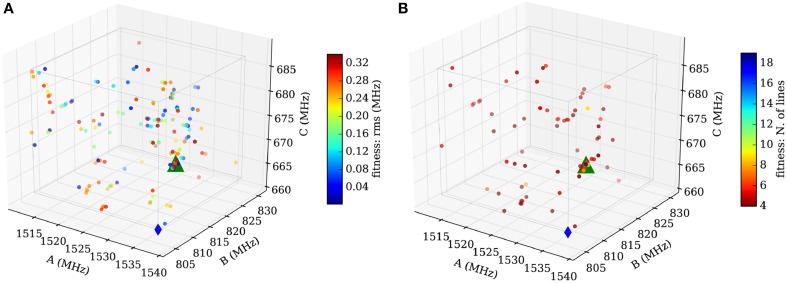
**Results for the subcube shaded red in Figure 9**. **(A)** Sample distribution after the fit employing SPFIT (step 2). The samples are color-coded using the value of the standard deviation provided by SPFIT. A subtle convergence toward the measured value (green triangle) is visible. **(B)** Sample distribution after evaluation of the fitting results (step 3). The samples are now color-coded using the number of assigned lines of the SPCAT prediction. The concentration of many samples at the measured rotational constants and the color-code of the samples indicates a correctly assigned spectrum (up to 18 assigned transitions). The comparison of **(A,B)** illustrates the necessity of the evaluation step 3. Good and bad fitting results are scattered throughout the volume in **(A)**, but **(B)** reveals that the samples far away from real values predict spectra which have little overlap with the measured one.

In summary, the best assignments from step one are fitted. Rules for choosing the proper transitions are mainly based on the intensities of the predicted and measured transitions. Diverging and unphysical fit results are directly eliminated, and only one sample is maintained for those which have converged to the same (A, B, C)-triple. The results are weighted using the root-mean-square error from the fit. The best fitting results within a sub-cube are plotted in Figure 10A.

#### Step 3: evaluation of the fitting results

In the final step, the fitting results from step two are reevaluated by a direct comparison of the predicted with the measured spectrum. This step is necessary, because the root-mean-square error is not a good check for the quality of a fit of three parameters including only four transitions. The overlap of the experimental transitions and the predicted transitions is assessed following the same prediction and assignment protocol as in step one, but with a much smaller tolerance (100 kHz in our case). The number of assigned transitions provides a reliable check of the quality of the assignment. Figure 10B shows the final result with a very good overlap of the automatically fitted samples and the manually fitted result (green triangle).

The assignment process depends heavily upon reliably measured and predicted intensities. In the case of untrustworthy experimental intensities or unavailable estimates of the dipole moments or the rotational temperature, it might be necessary to repeat the procedure employing different sets of dipole moment components and temperatures. In the present study, the routine was repeated three times, setting only one dipole moment component to be nonzero for the predictions in step one and two. However, for the evaluation of the fit in step three, all types of rotational transitions were included.

All four isomers present in the menthone/isomenthone mixture were found using as initial input the rotational constants from the B3LYP/aug-cc-pVTZ calculations. The results are compiled in the Supplementary Material, listing the ten best hits for each of the predicted isomers. Furthermore, the routine was also tested on the spectra (2−8 GHz) of two other molecules of the monoterpene family: carvone (843 peaks) and carvomenthenol (204 peaks). For these molecules the different conformers (two for carvone and three for carvomenthenol) were identified without any preprocessing to remove background lines. The parameters of the routine (bandwidth, cubesize, number of samples) were identical to the ones used for isomenthone eq-ax A. The computation time heavily depends on the line density of the spectrum under study. In the case of the spectra of carvone, carvomenthenol, and menthone, it took less than 1 h to run the routine using a single core on an Intel quad-core i5 processor (2.3 GHz).

The computer program is still limited to molecular spectra that can be fit using a rigid rotor Hamiltonian, but the implementation of common rotational spectroscopy effects such as nuclear quadrupole coupling or internal rotation is in progress. It is always possible to extend the parameter space and include more than just the rotational constants into the fit. But with a growing number of included parameters, the set of starting samples increases exponentially if the same parameter space density is maintained. More lines would need to be included into the fit in order to get reliable results, as expected with an increasing number of assignment possibilities. Hence, the required computation time grows rapidly. To counteract the increasing computation time, the different fitting parameters can be classified according to their impact on the fit. As a consequence, the seed set would no longer be uniformly distributed along each parameter axis but denser along high impact parameters. Parameters with a very small effect on the fit, such as the A-rotational constant in *a*-type spectra, can be kept constant throughout the fit. This approach should be feasible for spectra including nuclear quadrupole coupling splitting or centrifugal distortion.

## 4. Conclusions

We presented the microwave spectra of the structurally related monoterpenoids menthol, menthone, and isomenthone and an evaluation of their potential energy surfaces. For menthol, a single conformation was observed under the conditions of the supersonic expansion. The experimentally obtained rotational constants of the observed conformation agreed very well with those of the calculated lowest energy conformer.

In the broadband microwave spectrum of a mixture of the diastereomers menthone and isomenthone, three conformations of menthone and a single conformation of isomenthone were identified. Again, the comparison of the experimental rotational constants with the ab initio ones revealed a good match. The differences in the conformational landscape for menthone and isomenthone likely arise from the peculiar steric situation of the central cyclohexane ring, for which a chair configuration with sterically demanding substituents in the equatorial positions is preferred. Due to the arrangement of the stereogenic centers in isomenthone, one substituent (i.e., either the methyl or the isopropyl group) must be oriented axially, resulting in large shifts of the conformational landscape from menthone. Complications of assigning the dense microwave spectra manually led to a computer aided assignment routine, which was capable of identifying and fitting all components in the menthone mixture with inputs from ab initio calculated rotational constants.

### Conflict of interest statement

The authors declare that the research was conducted in the absence of any commercial or financial relationships that could be construed as a potential conflict of interest.
