# Supplementary material for: Exploring the conformational landscape of menthol, menthone, and isomenthone: a microwave study
Source: Front Chem. 2015 Mar 11;3:15. doi: 10.3389/fchem.2015.00015 (PMC4355985; doi:10.3389/fchem.2015.00015)
Supplement: Supplementary file 1 [file DataSheet1.PDF]

Supplementary Material for: Exploring the conformational landscape of menthol, menthone, and isomenthone: A microwave study

D. Schmitz,[1,2] V. A. Shubert,[1,2] T. Betz,[1,2] and M. Schnell[1,2,3,*]

[1] *Max Planck Institute for the Structure and Dynamics of Matter, Hamburg, Germany*

[2] *The Center for Free-Electron Laser Science, Hamburg, Germany*

[3] *The Hamburg Centre for Ultrafast Imaging, Universität Hamburg, Hamburg, Germany*

(Dated: December 23, 2014)

* Corresponding author: melanie.schnell@mpsd.mpg.de

CONTENTS

Linelists 2
Remarks 2
menthol 2
menthone A 5
menthone B 9
menthone C 14
isomenthone eq-ax A 17
Quantum chemical calculations 21
Remarks 21
Automated fitting results 27
Remarks 27

LINELISTS

Remarks

This document shows the line frequencies of all measured species. Most of the lines consist of only three points in the spectrum. Therefore we fitted the line assuming a triangular line profile. Thus we can give here more digits than our experimental accuracy for the observed frequencies. However, all fits were carried out using 20 kHz as experimental error for each line frequency. More points per line would be necessary to fit the line with a more realistic line profile, such as a Lorentzian function. The observed line frequencies (obs. freq.) and the calculated line frequencies (calc. freq.) are given in units of MHz. The difference between these two values (diff.) is given in units of kHz.

menthol

J''	K_a''	K_c''	J'	K_a'	K_c'	obs. freq.	calc. freq.	diff. (kHz)
2	1	2	1	1	1	2412.664	2412.6574	6.6

J''	K_a''	K_c''	J'	K_a'	K_c'	obs. freq.	calc. freq.	diff. (kHz)
1	1	0	0	0	0	2472.420	2472.4171	2.9
2	0	2	1	0	1	2522.642	2522.6484	-6.4
2	1	1	1	1	0	2651.212	2651.2100	2.0
4	2	3	4	1	3	2862.868	2862.8639	4.1
3	2	2	3	1	2	3088.656	3088.6539	2.1
4	0	4	3	1	2	3429.554	3429.5633	-9.3
3	1	3	2	1	2	3613.391	3613.3916	-0.6
3	0	3	2	0	2	3761.124	3761.1266	-2.6
3	2	2	2	2	1	3797.898	3797.8996	-1.6
3	2	1	2	2	0	3834.677	3834.6726	4.4
2	1	1	1	0	1	3857.656	3857.6601	-4.1
3	1	2	2	1	1	3970.769	3970.7671	1.9
4	2	2	4	1	4	4188.159	4188.1524	6.6
4	1	4	3	1	3	4808.031	4808.0261	4.9
4	0	4	3	0	3	4974.213	4974.2155	-2.5
4	2	3	3	2	2	5056.631	5056.6315	-0.5
4	3	2	3	3	1	5081.132	5081.1499	-17.9
4	3	1	3	3	0	5083.867	5083.8597	7.3
4	2	2	3	2	1	5146.265	5146.2619	3.1
4	1	3	3	1	2	5282.420	5282.4214	-1.4
3	1	2	2	0	2	5305.784	5305.7788	5.2
6	3	4	6	2	4	5310.434	5310.4372	-3.2
5	3	3	5	2	3	5505.829	5505.8442	-15.2

J''	K_a''	K_c''	J'	K_a'	K_c'	obs. freq.	calc. freq.	diff. (kHz)
4	3	2	4	2	2	5628.485	5628.4828	2.2
5	3	2	5	2	4	5824.788	5824.7851	3.0
2	2	0	1	1	0	5922.024	5922.0167	7.3
6	3	3	6	2	5	5930.395	5930.3949	0.1
5	1	5	4	1	4	5995.488	5995.4860	2.0
2	2	1	1	1	1	6032.008	6032.0077	0.3
7	3	4	7	2	6	6107.509	6107.5122	-3.2
5	0	5	4	0	4	6159.003	6158.9969	6.1
7	1	6	6	2	4	6268.570	6268.5716	-1.6
5	2	4	4	2	3	6309.207	6309.2040	3.0
5	3	3	4	3	2	6357.249	6357.2494	-0.4
5	3	2	4	3	1	6366.651	6366.6543	-3.3
5	2	3	4	2	2	6479.882	6479.8880	-6.0
5	1	4	4	1	3	6582.462	6582.4620	0.0
4	1	3	3	0	3	6827.077	6827.0737	3.4
3	2	1	2	1	1	7105.481	7105.4793	1.7
6	1	6	5	1	5	7175.377	7175.3717	5.3
6	0	6	5	0	5	7318.815	7318.8069	8.1
3	2	2	2	1	2	7417.244	7417.2498	-5.8
8	1	7	7	2	5	7444.982	7444.9868	-4.8
6	2	5	5	2	4	7554.182	7554.1817	0.3
6	4	3	5	4	2	7627.140	7627.1535	-13.5
6	3	4	5	3	3	7635.098	7635.1000	-2.0

J''	K_a''	K_c''	J'	K_a'	K_c'	obs. freq.	calc. freq.	diff. (kHz)
6	3	3	5	3	2	7659.789	7659.7916	-2.6
6	2	4	5	2	3	7830.509	7830.5071	1.9
6	1	5	5	1	4	7866.332	7866.3312	0.8
4	2	2	3	1	2	8280.975	8280.9742	0.9
7	1	7	6	1	6	8347.935	8347.9311	3.9
7	0	7	6	0	6	8462.001	8461.9937	7.3

menthone A

J''	K_a''	K_c''	J'	K_a'	K_c'	obs. freq.	calc. freq.	diff. (kHz)
1	1	1	0	0	0	2540.011	2540.0113	-0.3
2	0	2	1	0	1	2555.535	2555.5387	-3.7
3	0	3	2	1	2	2659.233	2659.2285	4.5
2	1	1	1	1	0	2670.118	2670.1237	-5.7
5	1	4	4	2	3	3231.269	3231.2677	1.3
3	1	3	2	1	2	3677.339	3677.3373	1.7
2	1	2	1	0	1	3713.166	3713.1660	0.0
3	0	3	2	0	2	3816.858	3816.8559	2.2
3	2	1	2	2	0	3869.700	3869.6998	0.2
3	1	2	2	1	1	4000.894	4000.8946	-0.6
4	0	4	3	1	3	4041.261	4041.2568	4.2
6	1	5	5	2	4	4790.395	4790.3940	1.0
3	1	3	2	0	2	4834.966	4834.9646	1.4
4	1	4	3	1	3	4895.932	4895.9354	-3.4

J''	K_a''	K_c''	J'	K_a'	K_c'	obs. freq.	calc. freq.	diff. (kHz)
4	0	4	3	0	3	5059.368	5059.3655	2.5
4	2	3	3	2	2	5119.199	5119.1941	4.9
4	3	2	3	3	1	5136.922	5136.9261	-4.1
4	3	1	3	3	0	5138.468	5138.4609	7.1
4	2	2	3	2	1	5184.192	5184.1900	2.1
4	1	3	3	1	2	5326.154	5326.1523	1.7
5	0	5	4	1	4	5424.836	5424.8357	0.3
4	1	4	3	0	3	5914.038	5914.0442	-6.2
5	1	5	4	1	4	6109.093	6109.1011	-8.1
9	2	7	8	3	6	6207.476	6207.4763	-0.3
5	0	5	4	0	4	6279.517	6279.5144	2.6
7	1	6	6	2	5	6375.441	6375.4419	-0.9
5	2	4	4	2	3	6390.694	6390.6927	1.3
5	3	3	4	3	2	6425.732	6425.7298	2.2
5	3	2	4	3	1	6431.078	6431.0735	4.5
2	2	1	1	1	0	6446.896	6446.8781	17.9
5	2	3	4	2	2	6516.575	6516.5729	2.1
2	2	0	1	1	1	6561.469	6561.4631	5.9
5	1	4	4	1	3	6643.447	6643.4468	0.2
6	0	6	5	1	5	6793.121	6793.1218	-0.8
5	1	5	4	0	4	6963.779	6963.7797	-0.7
6	1	6	5	1	5	7316.307	7316.3077	-0.7
6	0	6	5	0	5	7477.387	7477.3872	-0.2

J''	K_a''	K_c''	J'	K_a'	K_c'	obs. freq.	calc. freq.	diff. (kHz)
3	2	2	2	1	1	7620.027	7620.0320	-5.0
6	2	5	5	2	4	7656.702	7656.7159	-13.9
6	3	3	5	3	2	7730.438	7730.4421	-4.1
6	2	4	5	2	3	7865.586	7865.5841	1.9
6	1	5	5	1	4	7949.815	7949.8190	-4.0
8	1	7	7	2	6	7974.549	7974.5515	-2.5
3	2	1	2	1	2	7976.922	7976.9151	6.9
6	1	6	5	0	5	8000.570	8000.5730	-3.0
7	0	7	6	1	6	8133.922	8133.9214	0.6
6	1	5	6	0	6	2768.642	2768.6309	11.1
7	2	5	7	1	6	3283.209	3283.1992	9.9
6	2	4	6	1	5	3299.137	3299.1356	1.5
7	1	6	7	0	7	3353.303	3353.2874	15.6
8	2	6	8	1	7	3357.121	3357.1179	3.2
5	2	3	5	1	4	3383.371	3383.3704	0.6
4	2	2	4	1	3	3510.255	3510.2443	10.7
9	2	7	9	1	8	3538.170	3538.1696	0.5
3	2	1	3	1	2	3652.207	3652.2067	0.3
2	2	0	2	1	1	3783.400	3783.4015	-1.5
10	2	8	10	1	9	3839.879	3839.8834	-4.4
8	1	7	8	0	8	4043.429	4043.4269	2.1
2	2	1	2	1	2	4100.565	4100.5682	-3.2
3	2	2	3	1	3	4266.522	4266.5085	13.5

J''	K_a''	K_c''	J'	K_a'	K_c'	obs. freq.	calc. freq.	diff. (kHz)
5	2	4	5	1	5	4771.371	4771.3587	12.3
9	1	8	9	0	9	4822.140	4822.1375	2.5
6	2	5	6	1	6	5111.782	5111.7670	15.0
7	2	6	7	1	7	5510.592	5510.5811	10.9
10	1	9	10	0	10	5665.379	5665.3954	-16.4
8	3	5	8	2	6	5853.873	5853.8792	-6.2
8	2	7	8	1	8	5966.226	5966.2172	8.8
7	3	4	7	2	5	6082.679	6082.6899	-10.9
6	3	3	6	2	4	6269.281	6269.2853	-4.3
5	3	2	5	2	3	6404.425	6404.4272	-2.2
9	2	8	9	1	9	6475.774	6475.7843	-10.3
4	3	1	4	2	2	6489.917	6489.9266	-9.6
3	3	0	3	2	1	6535.678	6535.6557	22.3
3	3	1	3	2	2	6568.454	6568.4686	-14.6
4	3	2	4	2	3	6586.199	6586.2007	-1.7
5	3	3	5	2	4	6621.234	6621.2378	-3.8
6	3	4	6	2	5	6680.847	6680.8501	-3.1
7	3	5	7	2	6	6772.566	6772.5615	4.5
8	3	6	8	2	7	6903.647	6903.6499	-2.9
10	2	9	10	1	10	7035.122	7035.1276	-5.6
9	3	7	9	2	8	7080.686	7080.6867	-0.7
10	3	8	10	2	9	7309.132	7309.1462	-14.2
9	3	6	9	2	7	5603.064	5603.0678	-3.8

J''	K_a''	K_c''	J'	K_a'	K_c'	obs. freq.	calc. freq.	diff. (kHz)
5	1	4	5	0	5	2296.212	2296.1991	12.9
12	3	9	12	2	10	5018.681	5018.6814	-0.4
11	3	8	11	2	9	5153.226	5153.2210	5.0
14	3	11	14	2	12	5069.014	5069.0310	-17.0
10	3	7	10	2	8	5358.690	5358.6944	-4.4
11	3	8	10	4	7	5648.308	5648.3042	3.8
16	4	12	16	3	13	7006.558	7006.5381	19.9
12	3	10	12	2	11	7934.990	7934.9902	-0.2
13	4	9	13	3	10	8019.002	8019.0113	-9.3

menthone B

J''	K_a''	K_c''	J'	K_a'	K_c'	obs. freq.	calc. freq.	diff. (kHz)
2	0	2	1	0	1	2502.069	2502.0730	-4.0
3	0	3	2	1	2	2523.904	2523.9077	-3.7
1	1	1	0	0	0	2584.117	2584.1226	-5.6
5	1	4	5	0	5	2617.890	2617.8899	0.2
2	1	1	1	1	0	2642.744	2642.7464	-2.4
6	1	5	6	0	6	3209.647	3209.6463	0.7
6	2	4	6	1	5	3482.128	3482.1237	4.3
7	2	5	7	1	6	3511.044	3511.0315	12.5
5	2	3	5	1	4	3548.961	3548.9631	-2.1
3	1	3	2	1	2	3564.312	3564.3130	-1.0
8	2	6	8	1	7	3660.967	3660.9700	-3.0

J''	K_a''	K_c''	J'	K_a'	K_c'	obs. freq.	calc. freq.	diff. (kHz)
4	2	2	4	1	3	3680.191	3680.1883	2.7
2	1	2	1	0	1	3708.395	3708.3948	0.2
3	0	3	2	0	2	3730.229	3730.2295	-0.5
3	2	1	2	2	0	3803.815	3803.8064	8.7
3	2	1	3	1	2	3840.327	3840.3228	4.2
4	0	4	3	1	3	3892.208	3892.2110	-3.0
7	1	6	7	0	7	3936.070	3936.0656	4.4
9	2	7	9	1	8	3951.705	3951.7042	0.9
3	1	2	2	1	1	3958.105	3958.1043	0.7
2	2	0	2	1	1	3994.617	3994.6208	-3.8
6	1	5	5	2	4	4602.265	4602.2746	-9.6
4	1	4	3	1	3	4742.508	4742.5119	-3.9
3	1	3	2	0	2	4770.637	4770.6349	2.2
8	1	7	8	0	8	4781.612	4781.6038	8.2
4	2	3	4	1	4	4855.212	4855.2089	3.1
4	0	4	3	0	3	4932.621	4932.6164	4.6
4	2	3	3	2	2	5015.476	5015.4673	8.7
4	2	2	3	2	1	5105.530	5105.5260	4.0
5	2	4	5	1	5	5199.613	5199.6065	6.5
5	0	5	4	1	4	5255.082	5255.0886	-6.6
4	1	3	3	1	2	5265.659	5265.6605	-1.5
6	2	5	6	1	6	5615.737	5615.7289	8.1
9	1	8	9	0	9	5718.442	5718.4381	3.9

J''	K_a''	K_c''	J'	K_a'	K_c'	obs. freq.	calc. freq.	diff. (kHz)
9	3	6	9	2	7	5771.863	5771.8562	6.8
4	1	4	3	0	3	5782.915	5782.9173	-2.3
5	1	5	4	1	4	5913.362	5913.3632	-1.2
9	2	7	8	3	6	5949.639	5949.6464	-7.4
8	3	5	8	2	6	6057.059	6057.0580	1.1
7	2	6	7	1	7	6102.437	6102.4392	-2.2
5	0	5	4	0	4	6105.383	6105.3894	-6.4
7	1	6	6	2	5	6211.633	6211.6356	-2.6
5	2	4	4	2	3	6257.766	6257.7608	5.2
5	3	3	4	3	2	6306.141	6306.1394	1.6
5	3	2	4	3	1	6314.670	6314.6675	2.5
7	3	4	7	2	5	6335.086	6335.0863	-0.3
5	2	3	4	2	2	6430.626	6430.6119	14.1
5	1	4	4	1	3	6561.834	6561.8371	-3.1
6	3	3	6	2	4	6573.067	6573.0690	-2.0
6	0	6	5	1	5	6592.233	6592.2348	-1.8
2	2	1	1	1	0	6628.093	6628.0941	-1.1
8	2	7	8	1	8	6656.855	6656.8467	8.3
10	1	9	10	0	10	6712.486	6712.4881	-2.1
5	3	2	5	2	3	6751.742	6751.7451	-3.1
2	2	1	1	1	1	6759.496	6759.4946	1.4
5	1	5	4	0	4	6763.669	6763.6641	5.0
2	2	0	1	1	1	6768.771	6768.7677	3.3

J''	K_a''	K_c''	J'	K_a'	K_c'	obs. freq.	calc. freq.	diff. (kHz)
4	3	1	4	2	2	6867.682	6867.6895	-7.5
3	3	0	3	2	1	6930.691	6930.6986	-7.6
3	3	1	3	2	2	6976.346	6976.3504	-4.4
4	3	2	4	2	3	7000.937	7000.9466	-9.6
5	3	3	5	2	4	7049.322	7049.3252	-3.2
6	1	6	5	1	5	7076.323	7076.3222	0.9
6	3	4	6	2	5	7131.180	7131.1767	3.3
6	0	6	5	0	5	7250.508	7250.5094	-1.4
7	3	5	7	2	6	7256.269	7256.2747	-5.7
9	2	8	9	1	9	7274.238	7274.2340	4.0
8	3	6	8	2	7	7433.746	7433.7475	-1.5
6	2	5	5	2	4	7492.447	7492.4445	2.5
6	4	3	5	4	2	7565.383	7565.3808	2.2
6	4	2	5	4	1	7565.925	7565.9194	5.6
9	3	7	9	2	8	7671.426	7671.4373	-11.3
6	1	6	5	0	5	7734.599	7734.5968	2.3
3	2	2	2	1	1	7752.371	7752.3650	6.0
6	2	4	5	2	3	7775.428	7775.4264	1.6
8	1	7	7	2	6	7828.424	7828.4276	-3.6
6	1	5	5	1	4	7842.268	7842.2659	2.1
7	0	7	6	1	6	7891.304	7891.2989	5.1
10	2	9	10	1	10	7948.273	7948.2693	3.7
10	3	8	10	2	9	7975.380	7975.3838	-3.8

J''	K_a''	K_c''	J'	K_a'	K_c'	obs. freq.	calc. freq.	diff. (kHz)
6	3	4	5	3	3	7574.296	7574.2960	0.0
6	3	3	5	3	2	7596.744	7596.7503	-6.3
4	3	2	3	3	1	5040.070	5040.0635	6.5
4	3	1	3	3	0	5042.507	5042.5169	-9.9
2	1	2	1	1	1	2379.938	2379.9454	-7.4
5	1	4	4	2	3	3017.759	3017.7695	-10.5
6	5	2	5	5	1	7557.241	7557.2503	-9.3
3	2	1	2	1	2	8192.631	8192.6286	2.4
1	1	0	0	0	0	2715.511	2715.5231	-12.1
5	1	4	4	2	2	2881.647	2881.6487	-1.7
4	0	4	3	1	2	3104.216	3104.2183	-2.3
3	1	2	2	0	2	5558.629	5558.6276	1.4
7	1	6	6	2	4	5619.688	5619.6819	6.1
2	2	0	1	1	0	6637.369	6637.3672	1.8
3	2	1	2	1	1	7798.431	7798.4271	3.9
12	3	9	12	2	10	5284.386	5284.3886	-2.6
11	3	8	11	2	9	5345.280	5345.2885	-8.5
13	3	10	13	2	11	5368.172	5368.1885	-16.5
10	3	7	10	2	8	5520.466	5520.4598	6.2
9	3	7	9	2	7	5426.668	5426.6471	20.9
8	3	6	8	2	6	5878.199	5878.2029	-3.9
5	3	3	5	2	3	6740.352	6740.3534	-1.4
4	3	1	4	2	3	7003.809	7003.8103	-1.3

J''	K_a''	K_c''	J'	K_a'	K_c'	obs. freq.	calc. freq.	diff. (kHz)
9	1	8	8	2	6	7876.152	7876.1452	6.9
6	2	5	6	1	5	2890.178	2890.1699	8.1
5	2	4	5	1	4	3239.992	3239.9913	0.7
9	2	8	8	3	5	3526.003	3526.0012	1.8
2	2	1	2	1	1	3985.328	3985.3477	-19.7
4	3	2	4	2	2	6864.822	6864.8258	-3.8
3	3	1	3	2	1	6930.288	6930.2883	-0.3
5	3	2	5	2	4	7060.715	7060.7170	-2.0
11	1	10	11	0	11	7730.650	7730.6447	5.3

menthone C

J''	K_a''	K_c''	J'	K_a'	K_c'	obs. freq.	calc. freq.	diff. (kHz)
1	1	1	0	0	0	2707.520	2707.5186	1.5
3	1	3	2	1	2	3711.110	3711.1083	1.7
3	0	3	2	0	2	3823.759	3823.7632	-4.2
4	0	4	3	1	3	3846.168	3846.1615	6.5
2	1	2	1	0	1	3903.777	3903.7659	11.1
3	1	2	2	1	1	3960.048	3960.0510	-3.0
4	1	4	3	1	3	4944.254	4944.2539	0.1
4	0	4	3	0	3	5082.268	5082.2670	1.0
4	1	3	3	1	2	5275.742	5275.7437	-1.7
5	1	5	4	1	4	6174.332	6174.3333	-1.3
4	1	4	3	0	3	6180.358	6180.3595	-1.5

J''	K_a''	K_c''	J'	K_a'	K_c'	obs. freq.	calc. freq.	diff. (kHz)
5	0	5	4	0	4	6327.834	6327.8357	-1.7
5	2	3	4	2	2	6456.922	6456.9191	2.9
5	1	5	4	0	4	7272.423	7272.4257	-2.7
6	1	6	5	1	5	7400.887	7400.8990	-12.0
6	0	6	5	0	5	7558.946	7558.9453	0.7
8	2	6	8	1	7	3678.919	3678.9153	3.7
4	2	2	4	1	3	4053.355	4053.3506	4.4
4	2	3	4	1	4	4830.533	4830.5222	10.8
3	1	3	2	0	2	5059.870	5059.8687	1.3
5	2	4	5	1	5	5044.675	5044.6685	6.6
4	2	3	3	2	2	5114.303	5114.2983	4.7
4	2	2	3	2	1	5149.067	5149.0653	1.7
5	0	5	4	1	4	5229.741	5229.7433	-2.3
6	2	5	6	1	6	5303.512	5303.5157	-3.7
7	2	6	7	1	7	5607.367	5607.3702	-3.2
8	2	7	8	1	8	5956.083	5956.0852	-2.2
5	2	4	4	2	3	6388.481	6388.4795	1.5
5	3	3	4	3	2	6407.422	6407.4133	8.7
5	1	4	4	1	3	6587.442	6587.4450	-3.0
6	0	6	5	1	5	6614.353	6614.3553	-2.3
2	2	0	1	1	1	7012.831	7012.8317	-0.7
6	2	5	5	2	4	7659.746	7659.7462	-0.2
6	3	4	5	3	3	7692.340	7692.3511	-11.1

J''	K_a''	K_c''	J'	K_a'	K_c'	obs. freq.	calc. freq.	diff. (kHz)
6	2	4	5	2	3	7776.276	7776.2726	3.4
6	1	5	5	1	4	7893.810	7893.7991	10.9
7	0	7	6	1	6	7989.229	7989.2277	1.3
8	1	7	7	2	6	7181.528	7181.5223	5.7
6	3	3	6	2	4	7182.504	7182.5028	1.2
4	1	3	3	0	3	7009.830	7009.8276	2.4
5	2	3	5	1	4	3922.829	3922.8247	4.3
6	2	4	6	1	5	3805.305	3805.2982	6.9
5	3	2	5	2	3	7261.246	7261.2535	-7.5
7	3	5	7	2	6	7462.870	7462.8761	-6.1
3	1	2	2	0	2	5557.836	5557.8471	-11.1
4	2	3	4	1	3	4001.043	4001.0541	-11.1
4	3	1	4	2	2	7308.820	7308.8115	8.5
2	2	1	1	1	0	6926.303	6926.3055	-2.5
7	1	6	6	2	5	5625.179	5625.1846	-5.6
3	2	2	2	2	1	3837.781	3837.7762	4.8
7	2	5	7	1	6	3718.398	3718.4003	-2.3
10	3	7	10	2	8	6496.568	6496.5601	7.9
9	3	6	9	2	7	6716.868	6716.8638	4.2
8	3	5	8	2	6	6909.851	6909.8484	2.6
7	3	4	7	2	5	7066.154	7066.1513	2.7
8	3	6	8	2	7	7536.796	7536.7977	-1.7
8	3	6	8	2	6	6867.682	6867.6994	-17.4

J''	K_a''	K_c''	J'	K_a'	K_c'	obs. freq.	calc. freq.	diff. (kHz)

isomenthone eq-ax A

J''	K_a''	K_c''	J'	K_a'	K_c'	obs. freq.	calc. freq.	diff. (kHz)
7	2	5	7	1	6	2421.643	2421.6428	0.2
3	2	2	3	1	3	2814.835	2814.8301	4.9
6	1	5	6	0	6	2955.289	2955.2685	20.5
4	2	3	4	1	4	3116.476	3116.4720	4.0
5	2	4	5	1	5	3496.033	3496.0228	10.2
2	1	2	1	0	1	3549.577	3549.5783	-1.3
5	3	2	5	2	3	3591.925	3591.9282	-3.2
4	3	1	4	2	2	3772.591	3772.6036	-12.6
3	0	3	2	1	2	3780.224	3780.2210	3.0
2	1	1	1	0	1	3974.062	3974.0500	12.0
3	3	1	3	2	2	3976.526	3976.5277	-1.7
3	3	0	3	2	2	3978.125	3978.1090	16.0
4	3	2	4	2	3	4024.596	4024.5955	0.5
5	3	3	5	2	4	4116.151	4116.1581	-7.1
4	1	3	3	2	2	4210.205	4210.2031	1.9
6	3	4	6	2	5	4265.194	4265.1931	0.9
8	1	7	8	0	8	4535.913	4535.9019	11.1
8	3	6	8	2	7	4776.879	4776.8744	4.6
3	1	3	2	0	2	4829.440	4829.4463	-6.3
8	2	7	8	1	8	5045.511	5045.4987	12.3

J''	K_a''	K_c''	J'	K_a'	K_c'	obs. freq.	calc. freq.	diff. (kHz)
9	3	7	9	2	8	5149.208	5149.2029	5.1
7	4	3	7	3	4	5249.862	5249.8627	-0.7
4	0	4	3	1	3	5316.813	5316.8101	2.9
6	4	2	6	3	3	5391.963	5391.9682	-5.2
2	2	1	1	1	1	5418.751	5418.7465	4.5
2	2	0	1	1	1	5437.567	5437.5667	0.4
5	4	1	5	3	2	5471.886	5471.8911	-5.1
4	4	0	4	3	1	5511.023	5511.0266	-3.6
6	4	3	6	3	4	5511.286	5511.2859	0.1
5	4	2	5	3	3	5513.849	5513.8509	-1.9
4	4	1	4	3	2	5521.873	5521.8743	-1.3
7	4	4	7	3	5	5524.579	5524.5813	-2.3
4	1	4	3	1	3	5621.083	5621.0849	-1.9
9	2	8	9	1	9	5665.067	5665.0533	13.7
3	1	2	2	0	2	5676.811	5676.8084	2.6
6	2	4	5	3	3	5727.009	5726.9928	16.2
5	1	4	4	2	3	5963.409	5963.4017	7.3
4	1	4	3	0	3	6070.622	6070.6296	-7.6
4	2	2	3	2	1	6093.681	6093.6851	-4.1
4	1	3	3	1	2	6177.667	6177.6711	-4.1
3	2	2	2	1	1	6620.119	6620.1240	-5.0
3	2	1	2	1	1	6712.115	6712.1152	-0.2
5	0	5	4	1	4	6812.391	6812.3949	-3.9

J''	K_a''	K_c''	J'	K_a'	K_c'	obs. freq.	calc. freq.	diff. (kHz)
5	1	5	4	1	4	7000.465	7000.4651	-0.1
8	5	4	8	4	5	7035.368	7035.3633	4.7
7	5	2	7	4	3	7044.929	7044.9256	3.4
3	2	1	2	1	2	7136.597	7136.5870	10.0
5	1	5	4	0	4	7304.734	7304.7400	-6.0
4	1	3	3	0	3	7474.584	7474.5779	6.1
5	3	2	4	3	1	7503.565	7503.5643	0.8
6	1	5	5	2	4	7719.875	7719.8802	-5.2
4	2	3	3	1	2	7890.196	7890.1948	1.2
3	2	1	3	1	2	2059.461	2059.4592	1.8
6	2	4	6	1	5	2123.273	2123.2707	2.3
2	2	1	2	1	2	2591.502	2591.5186	-16.6
2	2	1	1	1	0	5277.254	5277.2559	-1.9
4	2	3	3	2	2	5922.727	5922.7268	0.2
6	0	6	5	1	5	8260.624	8260.6345	-10.5
2	2	0	1	1	0	5296.068	5296.0761	-8.1
9	4	5	9	3	6	4745.153	4745.1401	12.9
8	2	6	8	1	7	2893.736	2893.7425	-6.5
7	3	4	7	2	5	3154.153	3154.1489	4.2
9	4	5	9	3	6	4745.153	4745.1401	12.9
8	4	4	8	3	5	5031.492	5031.4830	9.0
8	4	5	8	3	6	5567.441	5567.4426	-1.6
10	3	8	10	2	9	5597.329	5597.3458	-16.8

J''	K_a''	K_c''	J'	K_a'	K_c'	obs. freq.	calc. freq.	diff. (kHz)
9	4	6	9	3	7	5655.069	5655.0687	0.3
10	5	5	10	4	6	6730.981	6730.9706	10.5
9	5	4	9	4	5	6888.199	6888.2025	-3.5
9	5	5	9	4	6	7008.358	7008.3595	-1.5
8	5	3	8	4	4	6986.344	6986.3449	-0.9
10	5	6	10	4	7	6989.116	6989.1163	-0.3
11	5	7	11	4	8	6989.434	6989.4378	-3.8
11	2	10	11	1	11	6989.570	6989.5776	-7.6
12	5	8	12	4	9	7023.793	7023.7986	-5.6
8	3	5	8	2	6	3010.712	3010.7060	6.0
11	4	8	11	3	9	6019.754	6019.7667	-12.7
10	1	9	10	0	10	6120.844	6120.8458	-1.8
11	1	10	11	0	11	6877.315	6877.3324	-17.4
8	3	5	7	4	4	7013.256	7013.2553	0.7

QUANTUM CHEMICAL CALCULATIONS

Remarks

The following tables present the results of the quantum chemical calculations at the M06-2X/6-311++G(d,p) level of theory for the five lowest energy menthol conformers and the three menthone conformers as well as the results of the quantum chemical calculations for the nine isomenthone conformers divided into three different groups according to the orientation of the mehtyl and isopropyl substituent: diequatorial (eq-eq), axial equatorial (ax-eq) and equatorial axial (eq-ax).

Table VI. Calculated relative zero-point energies, rotational constants, dipole moments, and torsional angles for the five lowest energy conformers of menthol using the M06-2X/6-311++G(d,p) method. We use the labeling scheme introduced by Moreno et al.

conformer	EQ1ext	EQ1int2	EQ1int	EQ3ext	EQ3int
ΔE (kJ/mol)	0.0	0.4	1.9/2.0/2.1	4.5/4.2/3.3	5.3/5.0/5.0
A (MHz)	1800	1773	1788	1983	1972
B (MHz)	703.8	693.8	703.1	675.8	675.5
C (MHz)	574.2	575.7	572.8	584.2	583.1
τ_1 (°)[a]	62.1	48.4	62.5	-158.1	-159.7
τ_2 (°)[a]	-176.4	-66.5	65.1	-175.2	-64.5
μ_a (D)[a]	1.4	0.8	-0.9	1.6	1.0
μ_b (D)[a]	-0.03	-1.2	-1.3	0.01	-1.1
μ_c (D)[a]	0.8	-0.8	0.9	0.8	-0.9
	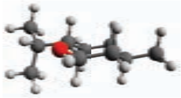	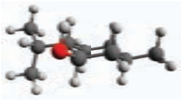	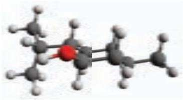	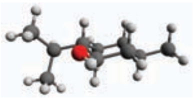	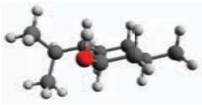

[a] Dipole moment components and dihedral angle are given for the (-)-enantiomer.

Table VII. Calculated relative zero-point energies, Boltzmann populations, rotational constants, dipole moments, and torsional angles for the three lowest energy conformers of menthone using the M06-2X/6-311++G(d,p) method.

	menthone A	menthone B	menthone C
ΔE (kJ/mol)	0.0	1.4	4.8
Population (%)[a]	53.2	34.9	11.8
A (MHz)	1969	2037	2125
B (MHz)	697.3	696.5	685.7
C (MHz)	590.2	564.5	601.8
μ_a (D)[b]	1.3	-1.3	1.8
μ_b (D)[b]	-2.6	-2.4	-2.2
μ_c (D)[b]	-0.5	1.2	-1.2
τ_1 (°)[b]	43.6	-48.5	178.1
	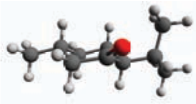	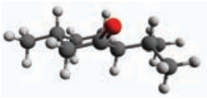	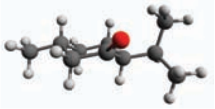

[a] Boltzmann distribution calculated at the sample reservoir temperature of 386 K.
[b] Dipole moment components and dihedral angle are given for the (-)-enantiomer.

Table VIII. Calculated relative zero-point energies, Boltzmann populations, rotational constants, dipole moments, and torsional angles for the three eq-eq conformers of isomenthone. The results were obtained using three different combinations of theoretical methods and basis sets and they are stated in this order: B3LYP/aug-cc-pVTZ / B3LYP/6-311++G(d,p) / MP2/6-311++G(d,p) / M06-2X/6-311++G(d,p).

	isomenthone eq-eq A	isomenthone eq-eq B	isomenthone eq-eq C
	B3LYP[b]/B3LYP[c]/MP2[c]/M06-2X[c]	B3LYP[b]/B3LYP[c]/MP2[c]/M06-2X[c]	B3LYP[b]/B3LYP[c]/MP2[c]/M06-2X[c]
ΔE (kJ/mol)	9.2/9.5/14.0/9.6	10.4/10.6/18.4/12.3	13.2/13.3/20.9/15.7
Population (%)[a]	2.5/2.2/0.7/1.5	2.1/1.6/0.2/0.7	0.7/0.7/0.1/0.2
A (MHz)	1935/1940/1950	1926/1918/1931/1939	2071/2063/2082/2093
B (MHz)	698.1/705.6/706.2	693.1/690.5/702.6/705.0	676.9/674.2/684.1/685.8
C (MHz)	576.4/589.1/584.4	586.9/584.5/595.4/594.5	600.2/598.0/611.2/608.8
μ_a (D)[d]	-1.3/-1.3/1.2/-1.3	1.3/1.4/1.3/1.4	1.8/1.8/1.6/1.8
μ_b (D)[d]	-2.3/-2.4/-2.2/-2.3	-2.6/-2.6/-2.5/-2.6	2.1/2.1/1.9/2.1
μ_c (D)[d]	1.4/1.5/-1.5/1.5	-0.6/-0.6/-0.7/-0.7	1.4/1.4/1.5/1.4
τ (°)[d]	-53.1/-53.2/-53.4/-52.8	46.2/46.4/48.4/49.1	-177.5/-177.4/-178.0/-176.8
	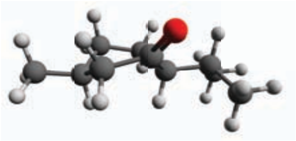	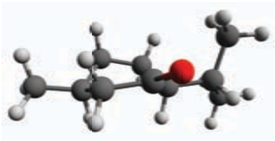	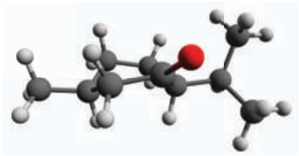

[a] Boltzmann distribution calculated at the sample reservoir temperature of 386 K.

[b] aug-cc-pVTZ basis set

[c] 6-311++G(d,p) basis set

[d] Dipole moment components and dihedral angle are given for the (-)-enantiomer.

Table IX. Calculated relative zero-point energies, Boltzmann populations, rotational constants, dipole moments, and torsional angles for the three ax-eq conformers of isomenthone. The results were obtained using three different combinations of theoretical methods and basis sets and they are stated in this order: B3LYP/aug-cc-pVTZ / B3LYP/6-311++G(d,p) / MP2/6-311++G(d,p) / M06-2X/6-311++G(d,p).

	isomenthone ax-eq A	isomenthone ax-eq B	isomenthone ax-eq C
	B3LYP[b]/B3LYP[c]/MP2[c]/M06-2X[c]	B3LYP[b]/B3LYP[c]/MP2[c]/M06-2X[c]	B3LYP[b]/B3LYP[c]/MP2[c]/M06-2X[c]
ΔE (kJ/mol)	2.7/2.5/3.7/0.0	2.7/2.5/4.4/0.2	5.5/5.0/7.4/3.7
Population (%)[a]	19.5/20.0/18.6/30.8	19.1/20.0/14.9/29.2	8.1/8.9/5.8/9.8
A (MHz)	1848/1839/1849/1847	1731/1722/1741/1740	1846/1837/1844/1849
B (MHz)	765.3/762.6/781.4/783.2	771.0/768.8/788.6/792.6	757.1/754.8/779.0/780.3
C (MHz)	624.8/622.5/641.0/640.4	674.1/672.0/693.4/695.1	685.1/683.3/706.1/705.9
μ_a (D)[d]	-1.1/-1.1/-0.9/-1.0	1.1/1.1/0.9/1.0	1.6/1.7/1.5/1.6
μ_b (D)[d]	2.4/2.5/2.2/2.4	-2.7/-2.7/-2.5/-2.7	-2.2/-2.3/-2.1/-2.2
μ_c (D)[d]	-1.4/-1.4/-1.4/-1.4	-0.7/-0.8/-0.7/-0.7	-1.3/-1.3/-1.3/-1.4
τ (°)[d]	-49.4/-49.4/-49.2/-49.0	42.6/42.9/42.0/43.4	-179.8/-179.8/177.1/177.1
	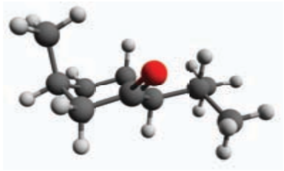	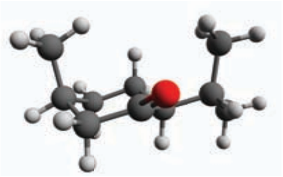	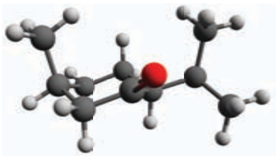

[a] Boltzmann distribution calculated at the sample reservoir temperature of 386 K.
[b] aug-cc-pVTZ basis set
[c] 6-311++G(d,p) basis set
[d] Dipole moment components and dihedral angle are given for the (-)-enantiomer.

Table X. Calculated relative zero-point energies, Boltzmann populations, rotational constants, dipole moments, and torsional angles for the three eq-ax conformers of isomenthone. The results were obtained using three different combinations of theoretical methods and basis sets and they are stated in this order: B3LYP/aug-cc-pVTZ / B3LYP/6-311++G(d,p) / MP2/6-311++G(d,p) / M06-2X/6-311++G(d,p).

	isomenthone eq-ax A	isomenthone eq-ax B	isomenthone eq-ax C
	B3LYP[b]/B3LYP[c]/MP2[c]/M06-2X[c]	B3LYP[b]/B3LYP[c]/MP2[c]/M06-2X[c]	B3LYP[b]/B3LYP[c]/MP2[c]/M06-2X[c]
ΔE (kJ/mol)	0.0/0.0/0.0/0.5	9.8/9.9/14.7/11.4	10.7/10.7/14.8/11.2
Population (%)[a]	44.6/43.2/58.4/26.0	2.1/2.0/0.6/0.9	1.6/1.5/0.6/0.9
A (MHz)	1538/1528/1516/1521	1641/1632/1643/1666	1715/1707/1644/1668
B (MHz)	804.5/802.2/824.6/827.4	771.1/768.3/782.4/781.1	769.8/766.7/810.5/808.3
C (MHz)	662.3/660.2/680.6/678.3	652.1/649.6/658.6/657.2	675.2/672.9/691.3/688.2
μ_a (D)[d]	-0.7/-0.7/-0.6/-0.7	0.7/0.7/0.6/0.8	1.3/1.3/-1.1/-1.3
μ_b (D)[d]	-3.1/-3.1/-2.9/-3.1	-3.0/-3.0/-2.8/-3.0	-2.8/-2.9/-2.7/-2.9
μ_c (D)[d]	-1.0/-1.0/-1.0/-1.0	0.1/0.1/0.1/0.1	0.1/0.1/-0.1/-0.1
τ (°)[d]	-64.5/-64.1/-64.0/-61.9	44.9/44.9/44.0/44.2	-176.9/-176.8/169.0/169.0
	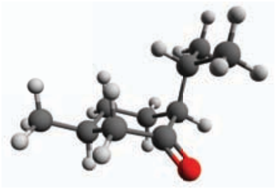	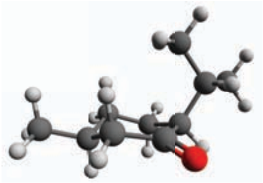	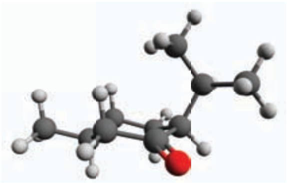

[a] Boltzmann distribution calculated at the sample reservoir temperature of 386 K.
[b] aug-cc-pVTZ basis set
[c] 6-311++G(d,p) basis set
[d] Dipole moment components and dihedral angle are given for the (-)-enantiomer.

AUTOMATED FITTING RESULTS

Remarks

The following tables summarize the ten best automated fitting results for all conformers of isomenthone and menthone. All experimentally observed conformers were assigned during the automated fitting process. The optimization of the isomenthone eq-eq conformers converged to the rotational constants of the menthone conformers. This convergence can be inhibited by removing the lines of the formerly assigned molecular species. The rotational constants A, B and C are given in MHz, N° is the number of assigned lines and μ defines the type of transition used in the fitting step (step two). The mean square deviation σ_{ms} is given in MHz^2 and calculated employing the following formula:

$$\sigma_{ms} = \frac{1}{N^2} \sum_{i=1}^{N} (\nu_i - f_i)^2$$

where ν_i are the predicted frequencies and f_i are the measured ones.

Table XI. Ten best automated fitting results using the calculated rotational constants of isomenthone eq-eq A (B3LYP/aug-cc-pVTZ) as starting values.

A	B	C	σ_{ms}	N°	μ
2021.850	693.537	562.137	3.2E-05	33	a
2021.896	693.550	562.132	8.9E-05	34	a
1953.514	694.522	586.574	3.4E-05	34	a
1953.514	694.522	586.574	3.4E-05	34	a
1953.379	694.510	586.582	2.4E-05	37	a
1953.400	694.514	586.578	3.6E-05	43	a
1953.401	694.520	586.576	4.9E-05	44	a
1953.436	694.519	586.564	3.1E-05	50	b
2022.009	693.550	562.131	4.3E-05	58	a
2021.999	693.538	562.135	2.0E-05	68	a

Table XII. Ten best automated fitting results using the calculated rotational constants of isomenthone eq-eq B (B3LYP/aug-cc-pVTZ) as starting values.

A	B	C	σ_{ms}	N°	μ
1953.493	694.516	586.576	2.4E-05	38	a
2022.081	693.537	562.135	5.2E-05	40	a
1953.471	694.525	586.573	4.9E-05	41	a
1953.408	694.520	586.575	4.1E-05	47	a
1953.452	694.520	586.575	3.0E-05	51	a
1953.451	694.520	586.575	3.0E-05	51	a
1953.422	694.513	586.582	2.0E-05	52	b
2022.008	693.538	562.134	2.3E-05	55	a
2021.964	693.536	562.136	3.0E-05	61	a
2021.965	693.536	562.136	3.5E-05	66	a

Table XIII. Ten best automated fitting results using the calculated rotational constants of isomenthone eq-eq C (B3LYP/aug-cc-pVTZ) as starting values.

A	B	C	σ_{ms}	N°	μ
1952.653	694.512	586.588	7.1E-05	28	a
2109.358	681.130	598.125	4.0E-05	28	a
1953.547	694.516	586.576	3.2E-05	31	a
2109.415	681.132	598.120	5.7E-05	32	b
2109.386	681.135	598.123	1.3E-05	34	a
1953.501	694.527	586.574	5.2E-05	35	a
1953.320	694.512	586.580	4.0E-05	35	a
1953.356	694.511	586.580	3.8E-05	37	a
1953.498	694.510	586.578	4.1E-05	38	a
1953.430	694.515	586.578	3.4E-06	54	a

Table XIV. Ten best automated fitting results using the calculated rotational constants of isomenthone eq-ax A (B3LYP/aug-cc-pVTZ) as starting values.

A	B	C	σ_{ms}	N°	μ
1463.895	813.682	668.896	3.7E-04	9	a
1537.287	803.964	650.316	2.7E-04	10	b
1518.892	811.005	671.149	4.0E-04	10	a
1542.375	780.033	679.898	2.5E-04	10	b
1600.239	800.236	656.142	2.7E-04	10	a
1531.863	801.651	655.617	4.8E-04	10	b
1519.958	826.978	680.046	2.5E-04	10	b
1544.963	815.468	640.884	2.1E-04	10	c
1535.233	812.852	671.459	2.7E-04	15	b
1535.280	812.916	671.443	1.1E-04	26	b

Table XV. Ten best automated fitting results using the calculated rotational constants of isomenthone eq-ax B (B3LYP/aug-cc-pVTZ) as starting values.

A	B	C	σ_{ms}	N°	μ
1622.322	765.681	648.388	1.9E-04	10	c
1638.075	756.387	636.368	2.8E-04	10	b
1399.408	786.839	671.031	1.3E-04	10	a
1620.913	758.304	636.578	3.3E-04	10	c
1629.937	765.336	636.930	2.4E-04	10	c
1630.828	749.528	672.439	1.6E-04	10	c
1654.801	739.471	655.284	3.3E-04	10	b
2044.831	799.406	651.847	2.1E-04	10	a
1631.719	781.620	673.301	1.2E-04	10	c
1661.867	758.067	662.308	1.9E-04	10	c

Table XVI. Ten best automated fitting results using the calculated rotational constants of isomenthone eq-ax C (B3LYP/aug-cc-pVTZ) as starting values.

A	B	C	σ_{ms}	N°	μ
1715.013	755.006	664.994	3.2E-05	10	b
1729.715	756.518	683.413	2.3E-04	10	b
1728.319	771.674	685.017	1.5E-04	10	b
1695.005	785.010	697.992	2.3E-04	10	c
1734.967	752.876	665.084	2.1E-04	10	b
1704.986	775.034	694.970	1.8E-04	11	c
1700.028	779.973	689.997	2.9E-04	11	c
1695.018	754.955	658.539	1.7E-04	11	c
1501.864	769.407	681.778	2.5E-04	11	a
1689.999	789.999	673.527	1.6E-04	11	c

Table XVII. Ten best automated fitting results using the calculated rotational constants of isomenthone ax-eq A (B3LYP/aug-cc-pVTZ) as starting values.

A	B	C	σ_{ms}	N°	μ
1835.930	785.317	599.792	3.4E-04	9	b
1840.693	788.091	603.849	2.8E-04	9	b
1392.729	749.284	632.172	3.1E-04	9	a
1832.287	751.919	634.978	3.3E-04	9	b
1781.179	787.613	651.470	1.7E-04	9	a
1866.844	750.975	644.998	2.4E-04	10	a
1850.171	746.871	629.824	2.6E-04	10	b
1841.172	754.867	646.417	4.6E-04	10	c
1857.740	770.438	601.089	1.1E-04	10	b
1927.817	749.741	630.383	3.0E-04	10	a

Table XVIII. Ten best automated fitting results using the calculated rotational constants of isomenthone ax-eq B (B3LYP/aug-cc-pVTZ) as starting values.

A	B	C	σ_{ms}	N°	μ
1717.042	762.955	682.014	2.1E-04	10	a
1728.319	771.674	685.017	1.5E-04	10	b
1722.635	771.639	685.968	2.6E-04	10	b
1747.504	792.500	687.725	2.4E-04	10	c
1634.254	745.706	650.178	2.8E-04	10	a
1746.248	753.733	651.246	7.5E-05	10	b
1725.415	776.553	669.020	3.6E-04	11	c
1804.933	789.482	655.166	2.5E-04	11	a
1723.634	776.996	669.104	3.1E-04	11	b
1716.021	763.999	691.568	1.1E-04	12	c

Table XIX. Ten best automated fitting results using the calculated rotational constants of isomenthone ax-eq C (B3LYP/aug-cc-pVTZ) as starting values.

A	B	C	σ_{ms}	N°	μ
1731.431	766.078	682.135	2.9E-04	9	a
1616.825	781.234	676.274	1.2E-04	9	a
1855.035	762.751	691.290	2.3E-04	9	c
1829.388	756.745	670.644	2.2E-04	9	b
1831.725	748.579	692.254	1.5E-04	10	b
1695.147	751.905	694.327	3.6E-04	10	a
1835.011	749.015	664.984	1.7E-04	10	b
1730.010	769.973	679.982	2.7E-04	11	a
1591.876	730.597	668.137	1.1E-04	11	a
1854.989	692.723	684.927	1.9E-04	14	b

Table XX. Ten best automated fitting results using the calculated rotational constants of menthone A (B3LYP/aug-cc-pVTZ) as starting values.

A	B	C	σ_{ms}	N°	μ
1953.562	694.525	586.576	7.0E-05	32	b
1953.303	694.511	586.577	4.7E-05	32	a
1953.538	694.513	586.577	4.7E-05	33	a
1953.530	694.515	586.576	5.7E-05	36	a
1953.392	694.504	586.592	4.6E-05	37	b
2022.032	693.537	562.135	1.8E-05	43	a
1953.400	694.514	586.578	3.6E-05	43	a
2021.901	693.536	562.136	4.3E-05	46	a
1953.447	694.509	586.578	2.7E-05	51	b
2022.013	693.537	562.136	3.0E-05	56	a

Table XXI. Ten best automated fitting results using the calculated rotational constants of menthone B (B3LYP/aug-cc-pVTZ) as starting values.

A	B	C	σ_{ms}	N°	μ
2022.859	693.538	562.128	5.6E-05	27	a
2021.727	693.536	562.139	1.4E-05	28	a
2021.858	693.515	562.142	9.9E-05	31	a
2021.834	693.537	562.137	3.6E-05	32	a
2021.896	693.550	562.132	8.9E-05	34	a
2022.006	693.571	562.140	9.4E-05	35	b
2022.006	693.571	562.140	9.3E-05	35	b
2022.047	693.545	562.132	3.7E-05	41	a
2021.937	693.538	562.136	2.1E-05	44	a
2022.009	693.537	562.134	2.6E-05	56	a

Table XXII. Ten best automated fitting results using the calculated rotational constants of menthone C (B3LYP/aug-cc-pVTZ) as starting values.

A	B	C	σ_{ms}	N°	μ
1952.867	694.514	586.584	5.5E-05	24	a
1953.202	694.527	586.574	8.4E-05	26	a
1953.609	694.512	586.577	1.6E-05	26	a
1953.181	694.525	586.574	6.8E-05	26	a
2109.391	681.151	598.126	1.6E-04	26	b
2109.308	681.134	598.124	7.8E-05	26	a
1953.747	694.512	586.579	2.7E-05	27	a
1953.571	694.517	586.575	3.2E-05	30	a
2109.394	681.135	598.123	7.6E-06	34	b
1953.397	694.523	586.574	6.4E-05	45	a
